# Supplementary material for: Adult skull bone marrow is an expanding and resilient haematopoietic reservoir
Source: Nature. 2024 Nov 13;636(8041):172–81. doi: 10.1038/s41586-024-08163-9 (PMC11618084; doi:10.1038/s41586-024-08163-9)
Supplement: Supplementary file 1 — Reporting Summary [file 41586_2024_8163_MOESM1_ESM.pdf]

nature portfolio

Corresponding author(s): Bong Ihn Koh
Ralf H. Adams

Last updated by author(s): Sep 3, 2024

Reporting Summary

Nature Portfolio wishes to improve the reproducibility of the work that we publish. This form provides structure for consistency and transparency in reporting. For further information on Nature Portfolio policies, see our Editorial Policies and the Editorial Policy Checklist.

Statistics

For all statistical analyses, confirm that the following items are present in the figure legend, table legend, main text, or Methods section.

n/a	Confirmed	
☐	☒	The exact sample size (*n*) for each experimental group/condition, given as a discrete number and unit of measurement
☐	☒	A statement on whether measurements were taken from distinct samples or whether the same sample was measured repeatedly
☐	☒	The statistical test(s) used AND whether they are one- or two-sided<br>*Only common tests should be described solely by name; describe more complex techniques in the Methods section.*
☐	☒	A description of all covariates tested
☐	☒	A description of any assumptions or corrections, such as tests of normality and adjustment for multiple comparisons
☐	☒	A full description of the statistical parameters including central tendency (e.g. means) or other basic estimates (e.g. regression coefficient) AND variation (e.g. standard deviation) or associated estimates of uncertainty (e.g. confidence intervals)
☐	☒	For null hypothesis testing, the test statistic (e.g. *F*, *t*, *r*) with confidence intervals, effect sizes, degrees of freedom and *P* value noted<br>*Give P values as exact values whenever suitable.*
☒	☐	For Bayesian analysis, information on the choice of priors and Markov chain Monte Carlo settings
☒	☐	For hierarchical and complex designs, identification of the appropriate level for tests and full reporting of outcomes
☒	☐	Estimates of effect sizes (e.g. Cohen's *d*, Pearson's *r*), indicating how they were calculated

Our web collection on statistics for biologists contains articles on many of the points above.

Software and code

Policy information about availability of computer code

Data collection	ZEN (Blue) 3.7 software (Carl Zeiss)<br>BD FACSDiva 9.1 software (BD Biosciences)<br>CFX Maestro 2.3 software (Bio-Rad)
Data analysis	ZEN (Black) 2.3 software (Carl Zeiss)<br>ImageJ 2.0.0 software (NIH)<br>IMARIS 10.0.1 software (Bitplane)<br>Prism 8.0 software (GraphPad)<br>FlowJo 10.3.0 (BD)

For manuscripts utilizing custom algorithms or software that are central to the research but not yet described in published literature, software must be made available to editors and reviewers. We strongly encourage code deposition in a community repository (e.g. GitHub). See the Nature Portfolio guidelines for submitting code & software for further information.

Data

Policy information about availability of data

All manuscripts must include a data availability statement. This statement should provide the following information, where applicable:

- Accession codes, unique identifiers, or web links for publicly available datasets
- A description of any restrictions on data availability
- For clinical datasets or third party data, please ensure that the statement adheres to our policy

The source data underlying all Figs. and Supplementary Figs. are provided as a Source Data file. All other data that support the findings of this study are available from the corresponding author upon reasonable request.

Research involving human participants, their data, or biological material

Policy information about studies with human participants or human data. See also policy information about sex, gender (identity/presentation), and sexual orientation and race, ethnicity and racism.

Reporting on sex and gender	Data from both sexes were used and consent to use this information was obtained.
Reporting on race, ethnicity, or other socially relevant groupings	All patients are Asian (Korean).
Population characteristics	9 females (ages 29-39 for young, 61-68 for old) and 9 males (ages 21-39 for young, 61-69 for old).
Recruitment	Patients who underwent CT for evaluation of small cerebral aneurysm from April to May 2023 were eligible. Patients were excluded if they had a previous history of surgery or radiation therapy to the head and neck, vascular or bone-related medical implants, or a suspicious disease other than small cerebral aneurysm.
Ethics oversight	Asan Medical Center, Seoul, Republic of Korea

Note that full information on the approval of the study protocol must also be provided in the manuscript.

Field-specific reporting

Please select the one below that is the best fit for your research. If you are not sure, read the appropriate sections before making your selection.

☒ Life sciences ☐ Behavioural & social sciences ☐ Ecological, evolutionary & environmental sciences

For a reference copy of the document with all sections, see nature.com/documents/nr-reporting-summary-flat.pdf

Life sciences study design

All studies must disclose on these points even when the disclosure is negative.

Sample size	No specific statistical methods were used to predetermine sample size.
Data exclusions	No samples were excluded from analysis.
Replication	Experiments were replicated at least once for all analyses and number of reproductions of each experimental finding is described in each figure legend. All attempts at experimental replication were successful.
Randomization	Animals from different cages, but within the same experimental group, were selected to assure randomization.
Blinding	N/A

Reporting for specific materials, systems and methods

We require information from authors about some types of materials, experimental systems and methods used in many studies. Here, indicate whether each material, system or method listed is relevant to your study. If you are not sure if a list item applies to your research, read the appropriate section before selecting a response.

Materials & experimental systems

n/a	Involved in the study	
☐	☒	Antibodies
☒	☐	Eukaryotic cell lines
☒	☐	Palaeontology and archaeology
☐	☒	Animals and other organisms
☒	☐	Clinical data
☒	☐	Dual use research of concern
☒	☐	Plants

Methods

n/a	Involved in the study	
☒	☐	ChIP-seq
☐	☒	Flow cytometry
☒	☐	MRI-based neuroimaging

Antibodies

Antibodies used

The following primary antibodies were used in the immunostaining of mouse samples: rat monoclonal anti-Endomucin (V.7C7) (Santa Cruz, Cat# sc-65495, 1:200 dilution), rabbit monoclonal anti-vATPaseB1/B2 (Abcam, Cat# 200839, 1:200 dilution), goat polyclonal anti-Osteopontin (R&D Systems, Cat# AF808, 1:200 dilution), goat polyclonal anti-CD31 (R&D, Cat# AF3628, 1:200 dilution), rabbit polyclonal anti-Caveolin 1 (Cell Signaling, Cat# 3238, 1:100), goat polyclonal anti-VEGF164 (R&D Systems, Cat# AF-493-NA, 1:200 dilution), rat monoclonal APC-conjugated anti-CD117 (c-Kit) (BD Biosciences, Cat# 553356, 1:100 dilution).

Species-specific secondary antibodies: Alexa Fluor 488 (Thermo Fischer Scientific, Cat# A21208), Alexa Fluor 594 (Thermo Fischer Scientific, Cat# A21209), Alexa Fluor 647 (Thermo Fischer Scientific, Cat# A31573 or Cat# A21447).

The following primary antibodies were used in in vivo immunostaining: rat monoclonal anti-CD31 (BD Biosciences, Cat# 553708. 1:10 dilution), rat monoclonal PE-conjugated anti-Endomucin (V.7C7)(Santa Cruz, Cat# 65495 PE, 1:10 dilution), rat monoclonal FITC-conjugated anti-CD45 (eBioscience, Cat# 11-0451-82, 1:10 dilution), hamster monoclonal FITC-conjugated anti-CD3e (eBioscience, Cat# 16-0031-82, 1:10 dilution), rat monoclonal PE-conjugated anti-CD45R/B220 (BD Biosciences, Cat# 553090, 1:10 dilution), rat monoclonal FITC-conjugated anti-CD11b (BD Biosciences, Cat# 553310, 1:10 dilution).

For lineage depletion, FACS sorting and analyses, the following antibodies were used: biotinylated rat monoclonal anti-hematopoietic lineage antibody cocktail (Miltenyi-Biotec, Cat# 130-092-613, 1:50 dilution), APC-conjugated rat monoclonal anti-CD117 (BD Biosciences, Cat# 553356, 1:100 dilution), FITC-conjugated rat monoclonal anti-CD117 (Biolegend, Cat# 105806, 1:100 dilution), FITC-conjugated rat monoclonal anti-Ly-6A/E (Sca-1) (eBioscience, Cat# 11-5981-85, 1:100 dilution), PerCP-Cy5.5-conjugated rat monoclonal anti-Ly-6A/E (Invitrogen, Cat# 45-5981, 1:100), APC-Cy7-conjugated hamster monoclonal anti-CD48 (BD Biosciences, Cat# 561242, 1:100 dilution), PE-conjugated rat monoclonal anti-CD150 (SLAM) (Biolegend, Cat# 115904, 1:100 dilution), Alexa Fluor 647-conjugated rat monoclonal anti-CD150 (Biolegend, Cat# 115918, 1:100 dilution), PE-Cy7-conjugated rat monoclonal anti-CD45 (eBioscience, Cat# 25-0451-82, 1:100 dilution), BV421-conjugated rat monoclonal anti-TER-119 (Biolegend, Cat# 116234, 1:100 dilution), FITC-conjugated rat monoclonal anti-CD71 (Biolegend, Cat# 113806, 1:100 dilution), Alexa Fluor 647-conjugated rat monoclonal anti-CD31 (BD Biosciences, Cat# 553708, conjugation described above, 1:200 dilution), PE-conjugated rat monoclonal anti-Endomucin (Santa Cruz, Cat# 65495 PE, 1:100 dilution), PE-Cy7-conjugated rat monoclonal anti-CD16/32 (eBioscience, Cat# 25-0161, 1:100 dilution), eFluor 450-conjugated rat monoclonal anti-CD34 (eBioscience, Cat# 48-0341, 1:100 dilution), PE-conjugated rat monoclonal anti-CD127 (eBioscience, Cat# 12-1271, 1:100 dilution), BV711-conjugated rat monoclonal anti-CD41 (BD Biosciences, Cat# 740712, 1:100 dilution), PE-conjugated rat monoclonal anti-CD105 (eBioscience, Cat# 12-1051-82, 1:100 dilution), APC-conjugated hamster monoclonal anti-CD3e (eBioscience, Cat# 17-0031, 1:100 dilution), PE-conjugated rat monoclonal anti-CD45R/B220 (BD Biosciences, Cat# 553090, 1:100 dilution), FITC-conjugated rat monoclonal anti-CD11b (BD Biosciences, Cat# 553310, 1:100 dilution), Alexa Fluor 405-(Invitrogen, Cat# S32351, 1:100 dilution) or APC-Cy7-(BD Biosciences, Cat# 554063, 1:100 dilution) conjugated streptavidin secondary antibody.

Validation

All the antibodies were validated for the species and applications (immunohistochemistry and FACS) by the corresponding manufacturer, which is described in the manufacturer's website. Our usage is described in the Methods section of the manuscript.

Animals and other research organisms

Policy information about studies involving animals; ARRIVE guidelines recommended for reporting animal research, and Sex and Gender in Research

Laboratory animals

C57BL/6J mice were used for all experiments involving wild-type mice. Flk1-GFP reporter (Xu et al., 2010), Vav1-Cre, ROSA26-mTmG reporter, ROSA26-CAG-loxP-stop-loxP-KikGR knock-in mice were transferred, established, and bred in the SPF animal facility at Max Planck Institute for Molecular Biomedicine. All of these mice were maintained in the C57BL/6 background. Mice at the age of 10-14 weeks, 31-37 weeks, 52-75 weeks and >95 weeks were chosen for young, middle-aged, old and geriatric groups, respectively.

Mice were kept in individually ventilated cages (IVC), with constant access to food and water under a 12h light and 12h dark cycle regime. Air flow, temperature (21-22[illegible]C) and humidity (55-60%) were controlled by an air management system. Animals were checked daily and maintained in specific pathogen-free (SPF) conditions. Sufficient nesting material and environmental enrichment was provided.

Wild animals

The study did not involve wild animals.

Reporting on sex

Indicate if findings apply to only one sex; describe whether sex was considered in study design, methods used for assigning sex.

Reporting on sex | *Provide data disaggregated for sex where this information has been collected in the source data as appropriate; provide overall numbers in this Reporting Summary. Please state if this information has not been collected. Report sex-based analyses where performed, justify reasons for lack of sex-based analysis.*

Field-collected samples | The study did not involve samples collected from the field.

Ethics oversight | All animal experiments were performed according to the institutional guidelines and laws, approved by local animal ethical committee and were conducted at the Max Planck Institute for Molecular Biomedicine (84-02.04.2016.A160, 81-02.04.2018.A171, 81-02.04.2020.A212, 81-02.04.2020.A416 and 81-02.04.2022.A198), Universitätsmedizin Berlin (G0220/17), Georg-Speyer-Haus (F123/2017) and the University Medical Center Mainz Institute of Transfusion Medicine (G23-1-067 A1TE) under the indicated permissions granted by the Landesamt für Natur, Umwelt und Verbraucherschutz (LANUV) of North Rhine-Westphalia, the State Office for Health and Social Affairs Berlin, Regierungspräsidium Darmstadt and the Landesuntersuchungsamt Rheinland-Pfalz, Germany.

Note that full information on the approval of the study protocol must also be provided in the manuscript.

Flow Cytometry

Plots

Confirm that:

☒ The axis labels state the marker and fluorochrome used (e.g. CD4-FITC).

☒ The axis scales are clearly visible. Include numbers along axes only for bottom left plot of group (a 'group' is an analysis of identical markers).

☒ All plots are contour plots with outliers or pseudocolor plots.

☒ A numerical value for number of cells or percentage (with statistics) is provided.

Methodology

Sample preparation | Mice from each age group were euthanized and skull and femur were harvested. Skulls were chopped with scissors in FACS buffer before crushed with mortar and pestle; femurs were crushed without chopping. BM stromal samples were dissociated with Collagenase I (Gibco, Cat# 17100-017, 2 mg/ml) and Collagenase IV (Gibco, Cat# 17104-019, 2 mg/ml) in PBS for 20 minutes at 37?C with intermittent shaking. Cell suspensions were strained through a 40 ?m mesh filter, resuspended in RBC lysing buffer (when applicable) and washed with FACS buffer.

Instrument | FACSAria Fusion (BD Biosciences), FACSymphony A5 Cell Analyzer (BD Biosciences)

Software | FACSDiva 8.0.2 software (BD Biosciences), FACSDiva 9.1 software (BD Biosciences)

Cell population abundance | Sorted LIN-negative cells were 1.5~2.0% of single cells. Purity was achieved at 95~98% and was confirmed by immediate analysis on a FACS analyzer.

Gating strategy | Preliminary FSC/SSC gating was established based on the exclusion of cellular debris and erythrocytes. Single cells were gated based on a single linear cluster within FSC-W vs. FSC-A. Positive populations (>10^3 fluorescence intensity) were gated based on clear population separation from the negative population (<10^2 fluorescence intensity).

☒ Tick this box to confirm that a figure exemplifying the gating strategy is provided in the Supplementary Information.
