# Supplementary material for: Adult skull bone marrow is an expanding and resilient haematopoietic reservoir
Source: Nature. 2024 Nov 13;636(8041):172–81. doi: 10.1038/s41586-024-08163-9 (PMC11618084; doi:10.1038/s41586-024-08163-9)

Adult skull bone marrow is an expanding and resilient hematopoietic reservoir

Bong Ihn Koh[1,#], Vishal Mohanakrishnan[1], Hyun-Woo Jeong[2], Hongryeol Park[1], Kai Kruse[3], Young Jun Choi[4], Melina Nieminen-Kelhä[5], Rahul Kumar[6], Raquel S. Pereira[7], , Susanne Adams[1], Hyuek Jong Lee[8], M. Gabriele Bixel[1], Peter Vajkoczy[5], Daniela S. Krause[6], Ralf H. Adams[1,#]

Supplementary Data File 1

Flow cytometric analyses of skull or femur BM in aging, pregnancy, stroke, CML, PTH treatment, Young *versus* Old transplantation, PGE2 treatment, AMD3100 treatment, *pLIVE-Vegfa* overexpression and DC101 treatment

<u>Table of contents</u>

Flow Cytometric Analysis of Aging Skull BM

Flow Cytometric Analysis of Aging Femur BM

Flow Cytometric Analysis of Skull BM in Pregnancy

Flow Cytometric Analysis of Femur BM in Pregnancy

Flow Cytometric Analysis of Skull BM in Stroke

Flow Cytometric Analysis of Femur BM in Stroke

Flow Cytometric Analysis of Skull BM in Chronic Myeloid Leukemia (CML)

Flow Cytometric Analysis of Femur BM in Chronic Myeloid Leukemia (CML)

Flow Cytometric Analysis of Skull BM after PTH Treatment

Flow Cytometric Analysis of Femur BM after PTH Treatment

Flow Cytometric Analysis of Skull BM after Young *versus* Old Donor Transplantation

Flow Cytometric Analysis of Femur BM after Young *versus* Old Donor Transplantation

Flow Cytometric Analysis of Skull BM after PGE2 Treatment

Flow Cytometric Analysis of Femur BM after PGE2 Treatment

Flow Cytometric Analysis of Skull BM after AMD3100 Treatment

Flow Cytometric Analysis of Femur BM after AMD3100 Treatment

Flow Cytometric Analysis of Skull BM with *pLIVE-Vegfa* Overexpression

Flow Cytometric Analysis of Femur BM with *pLIVE-Vegfa* Overexpression

Flow Cytometric Analysis of Skull BM after DC101 Treatment

Flow Cytometric Analysis of Femur BM after DC101 Treatment

Adult skull bone marrow is an expanding and resilient hematopoietic reservoir

Bong Ihn Koh[1,#], Vishal Mohanakrishnan[1], Hyun-Woo Jeong[2], Hongryeol Park[1], Kai Kruse[3], Young Jun Choi[4], Melina Nieminen-Kelhä[5], Rahul Kumar[6], Raquel S. Pereira[7], , Susanne Adams[1], Hyuek Jong Lee[8], M. Gabriele Bixel[1], Peter Vajkoczy[5], Daniela S. Krause[6], Ralf H. Adams[1,#]

Supplementary Data File 1

Abbreviations

EC: Endothelial Cell

$Emcn^{Hi}$: Endomucin-High

LT-HSC: Long-Term Hematopoietic Stem Cell

ST-HSC: Short-Term Hematopoietic Stem Cell

MPP2: Multi-Potent Progenitor 2

MPP3: Multi-Potent Progenitor 3

LSK: Lin^{-} $Sca1^{+}$ $cKit^{+}$

CLP: Common Lymphoid Progenitor

CMP: Common Myeloid Progenitor

GMP: Granulocyte-Monocyte Progenitor

MEP: Megakaryocyte-Erythrocyte Progenitor

MkP: Megakaryocyte Progenitor

Pre-GM: Pre-Granulocyte-Monocyte

Pre-CFU-E: Pre-Colony-Forming Unit-Erythroid

Pre-Meg-E: Pre-Megakaryocyte-Erythroid

CFU-E: Colony-Forming Unit-Erythroid

Pro-Ery: Pro-Erythroid

Flow Cytometric Analysis of Aging Skull BM: Young (Y), Middle-Aged (M), Old (O), Geriatric (G)

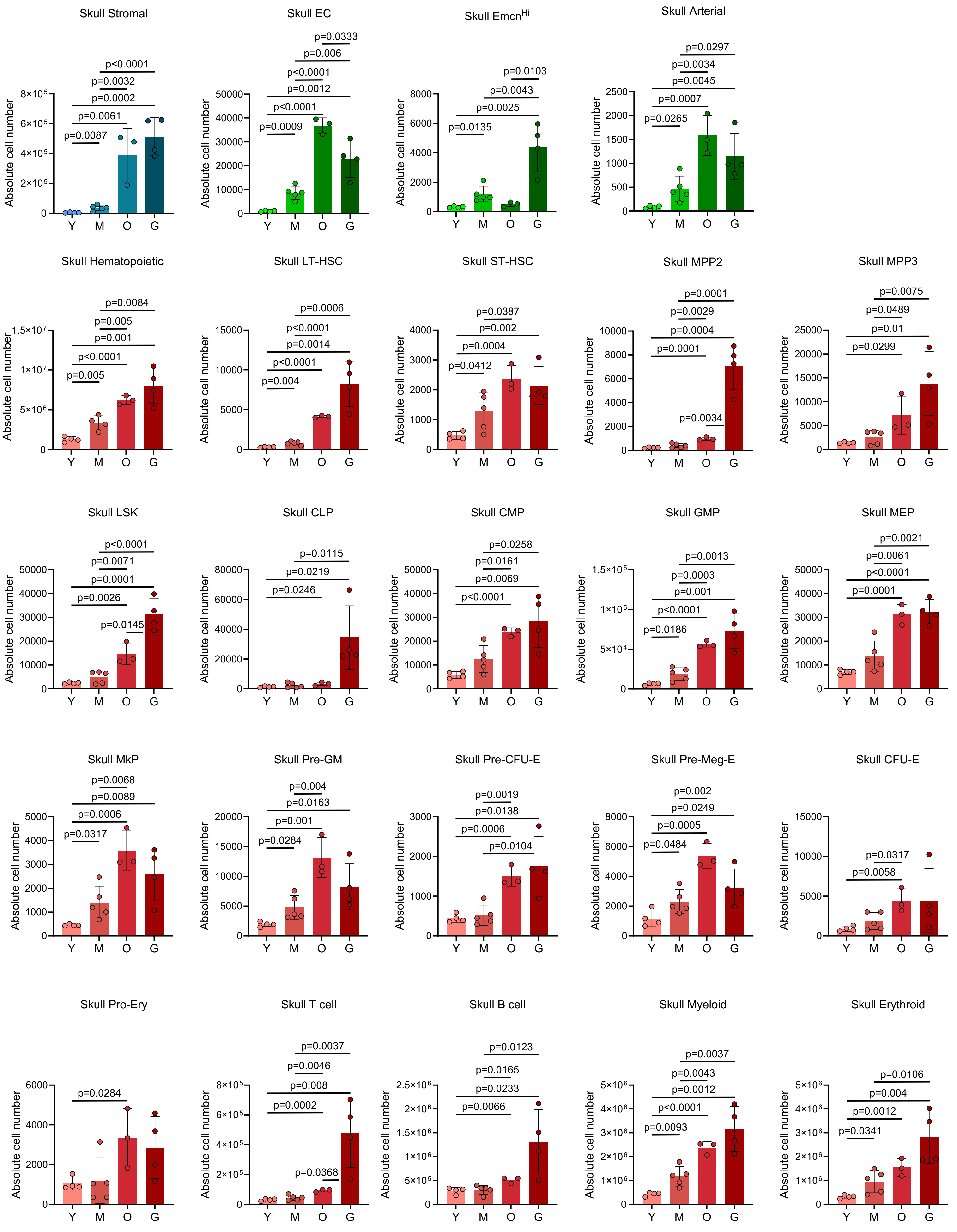

Flow Cytometric Analysis of Aging Femur BM: Young (Y), Middle-Aged (M), Old (O), Geriatric (G)

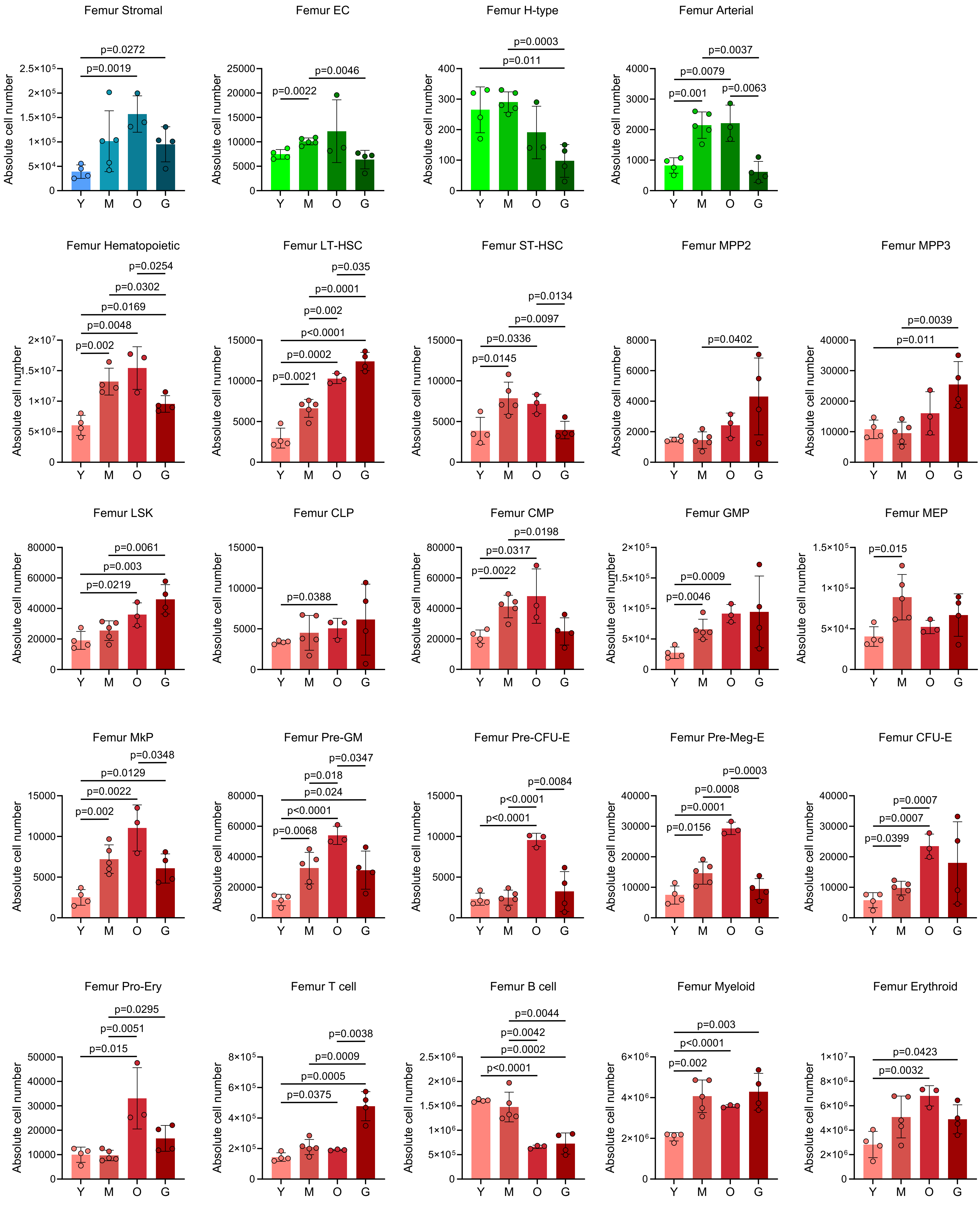

Flow Cytometric Analysis of Skull BM in Pregnancy (Dpp2: 2 Days post-partum)

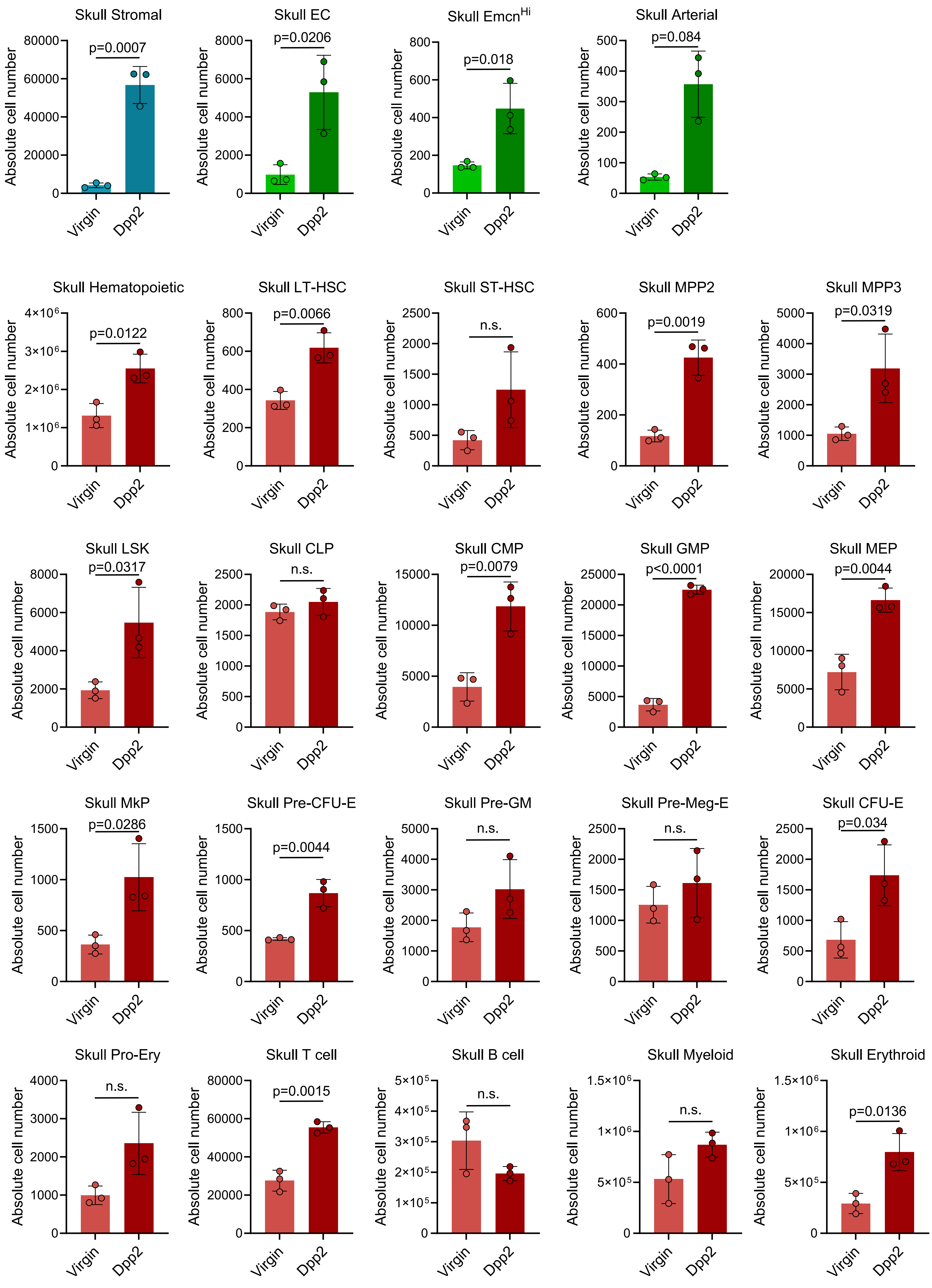

Flow Cytometric Analysis of Femur BM in Pregnancy (Dpp2: 2 Days post-partum)

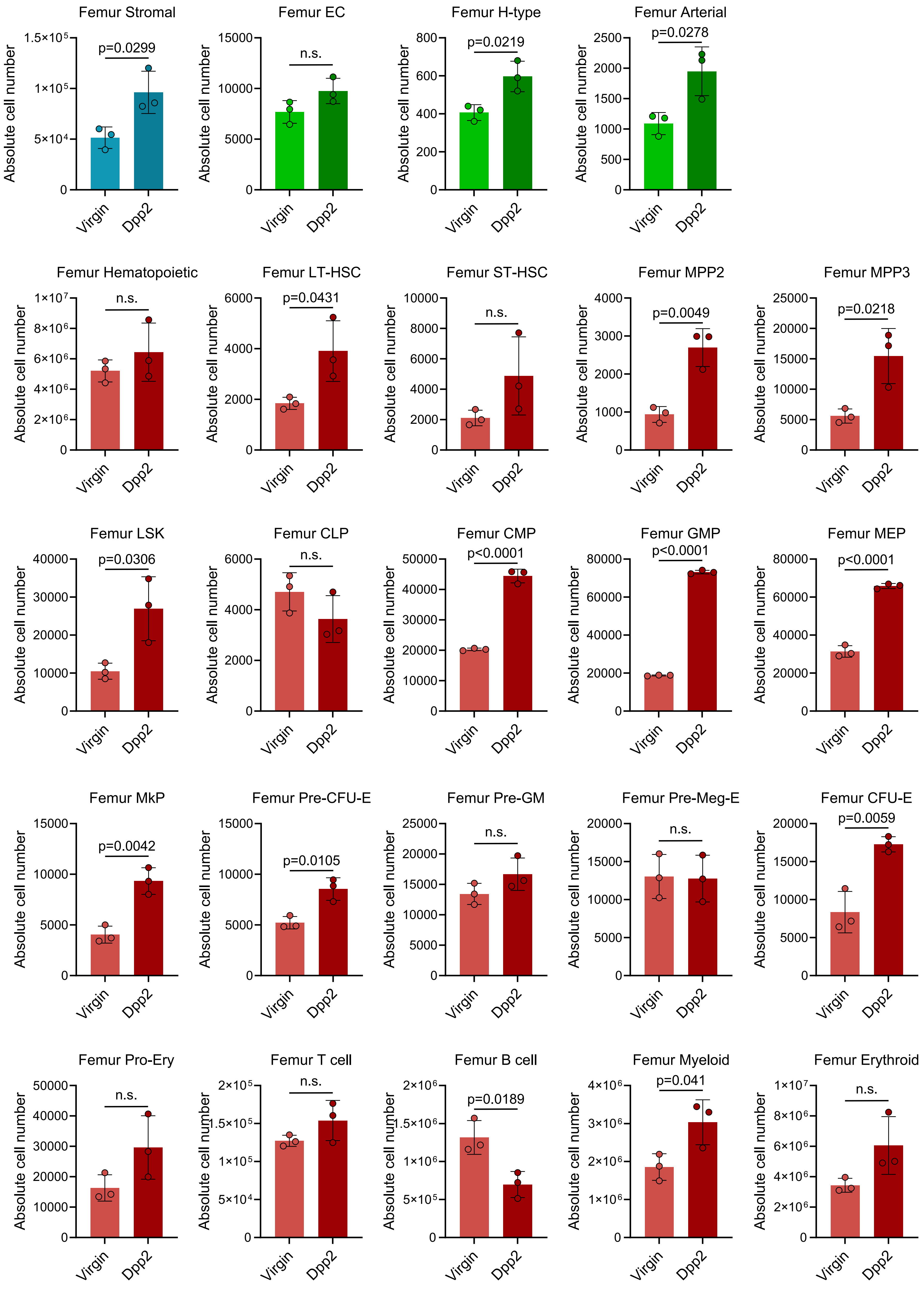

Flow Cytometric Analysis of Skull BM in Stroke (tMCAO: transient Mid-Cerebral Artery Occlusion)

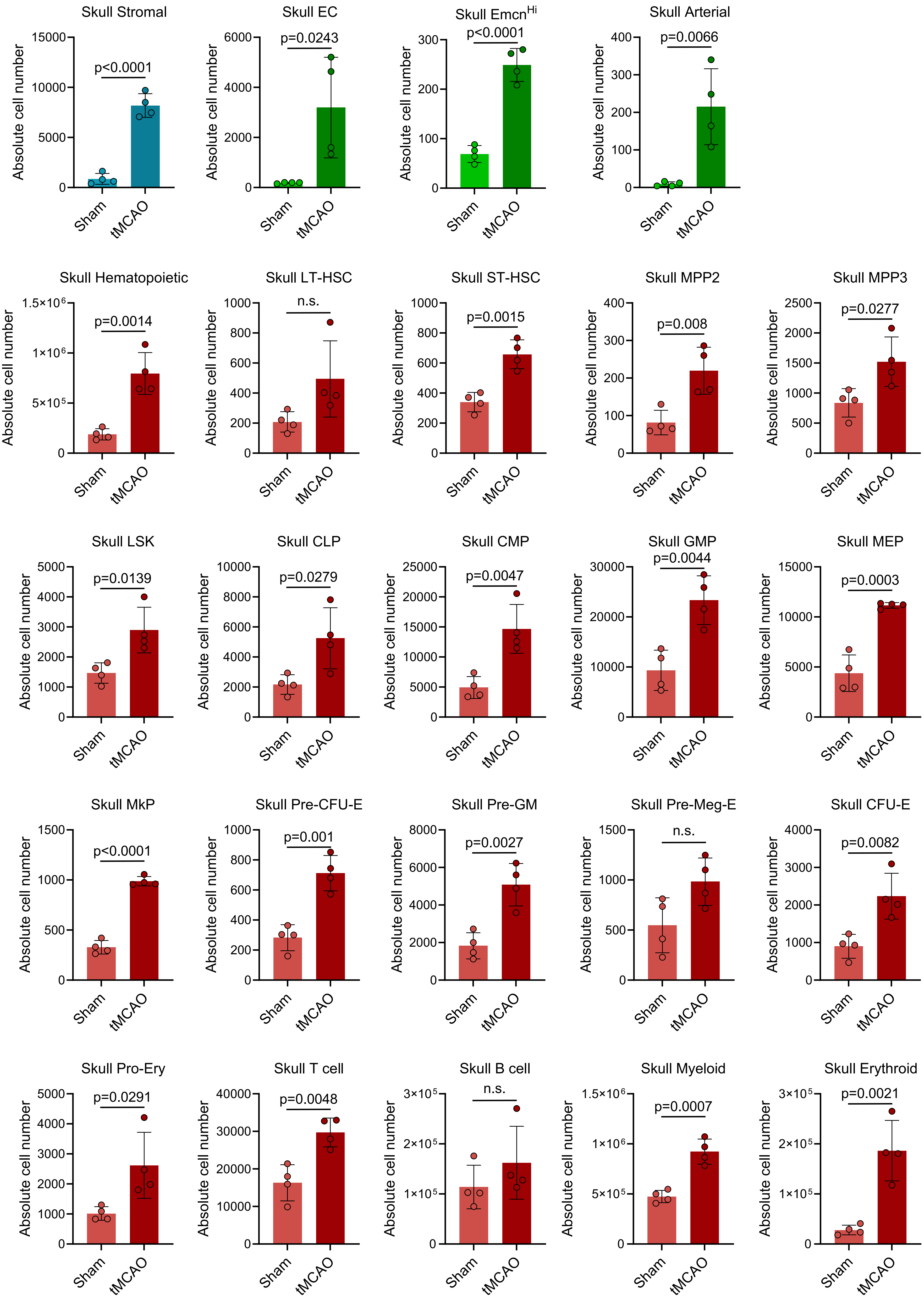

Flow Cytometric Analysis of Femur BM in Stroke (tMCAO: transient Mid-Cerebral Artery Occlusion)

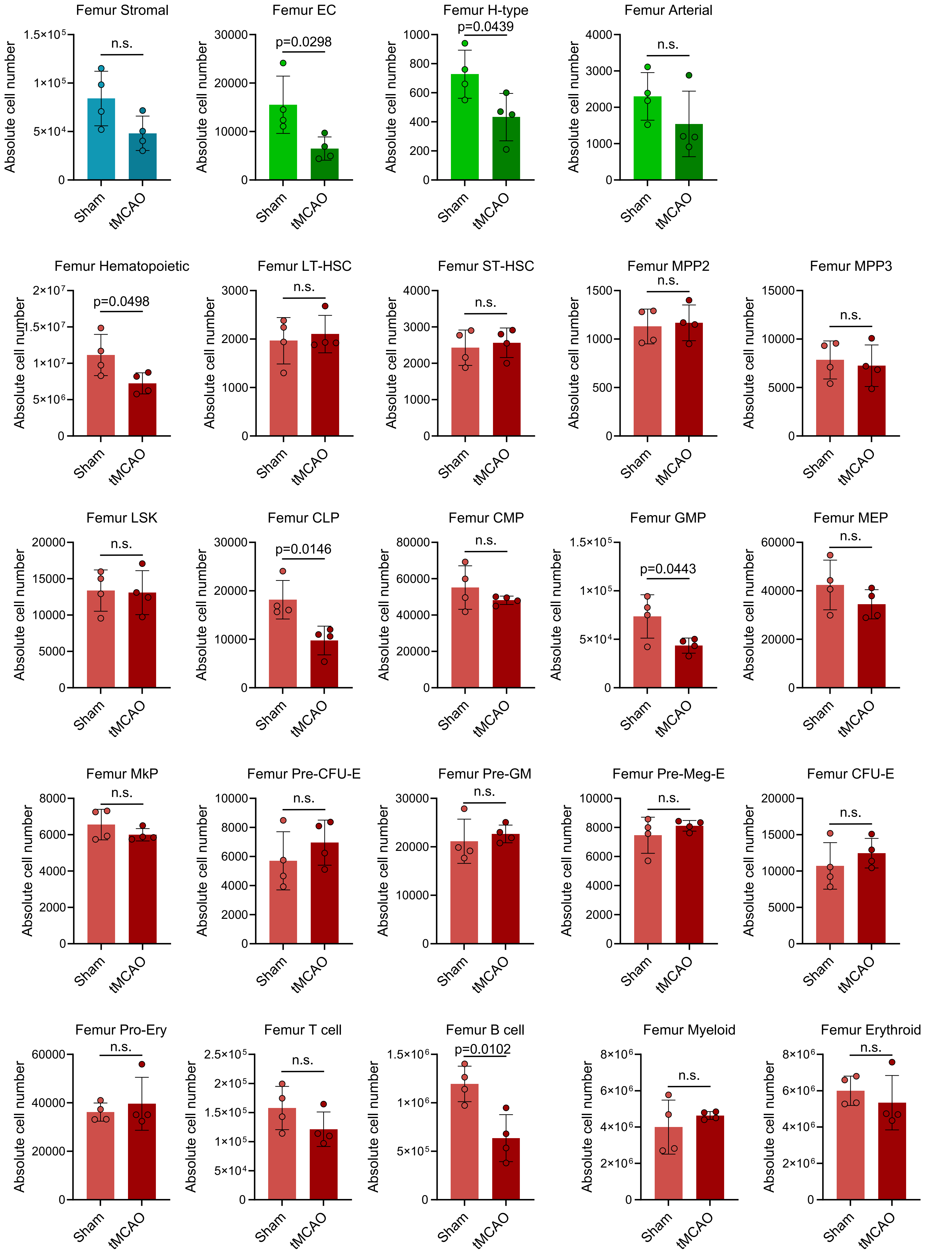

Flow Cytometric Analysis of Skull BM in Chronic Myeloid Leukemia (CML)

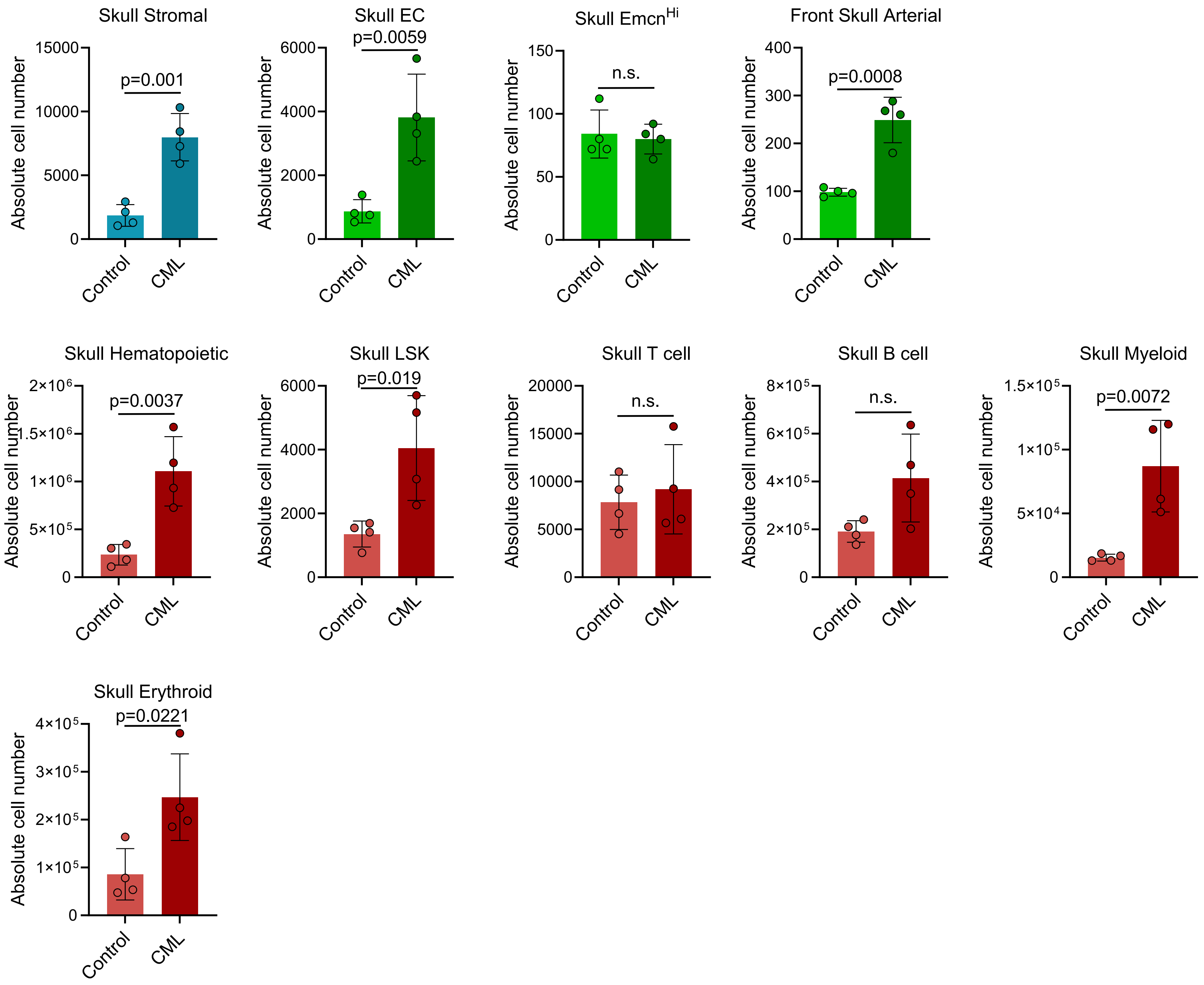

Flow Cytometric Analysis of Femur BM in Chronic Myeloid Leukemia (CML)

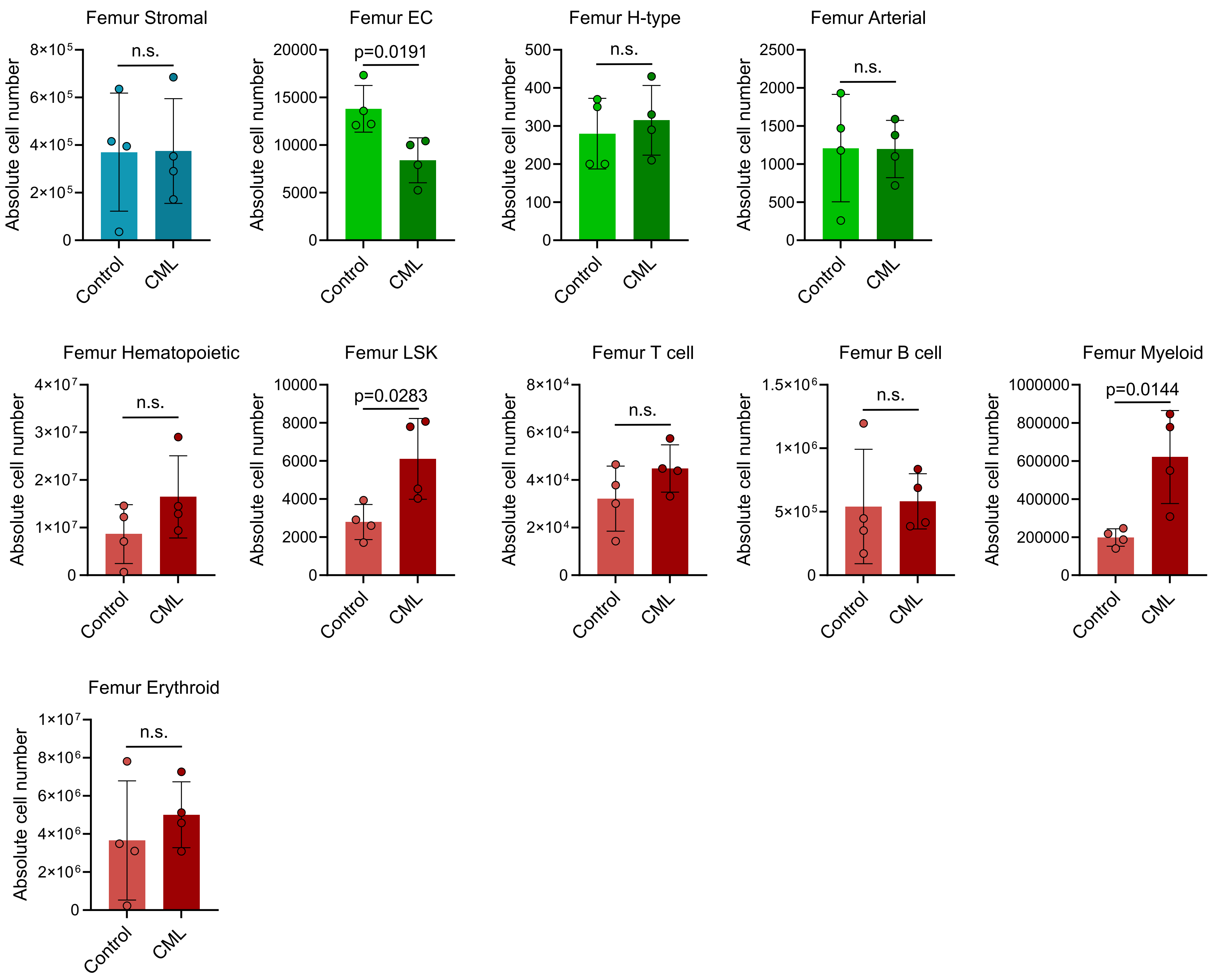

Flow Cytometric Analysis of Skull BM after Parathyroid Hormone (PTH) Treatment

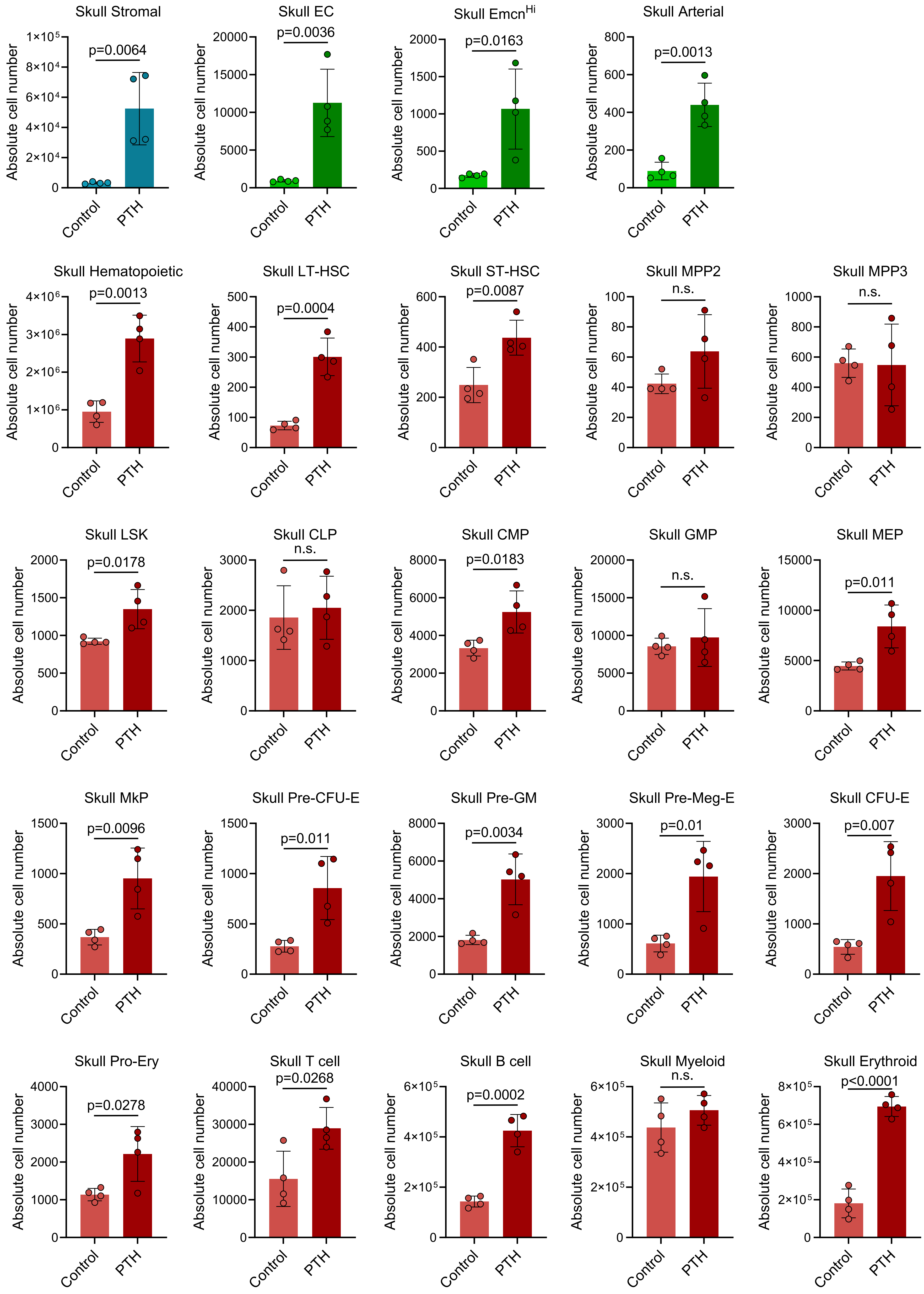

Flow Cytometric Analysis of Femur BM after Parathyroid Hormone (PTH) Treatment

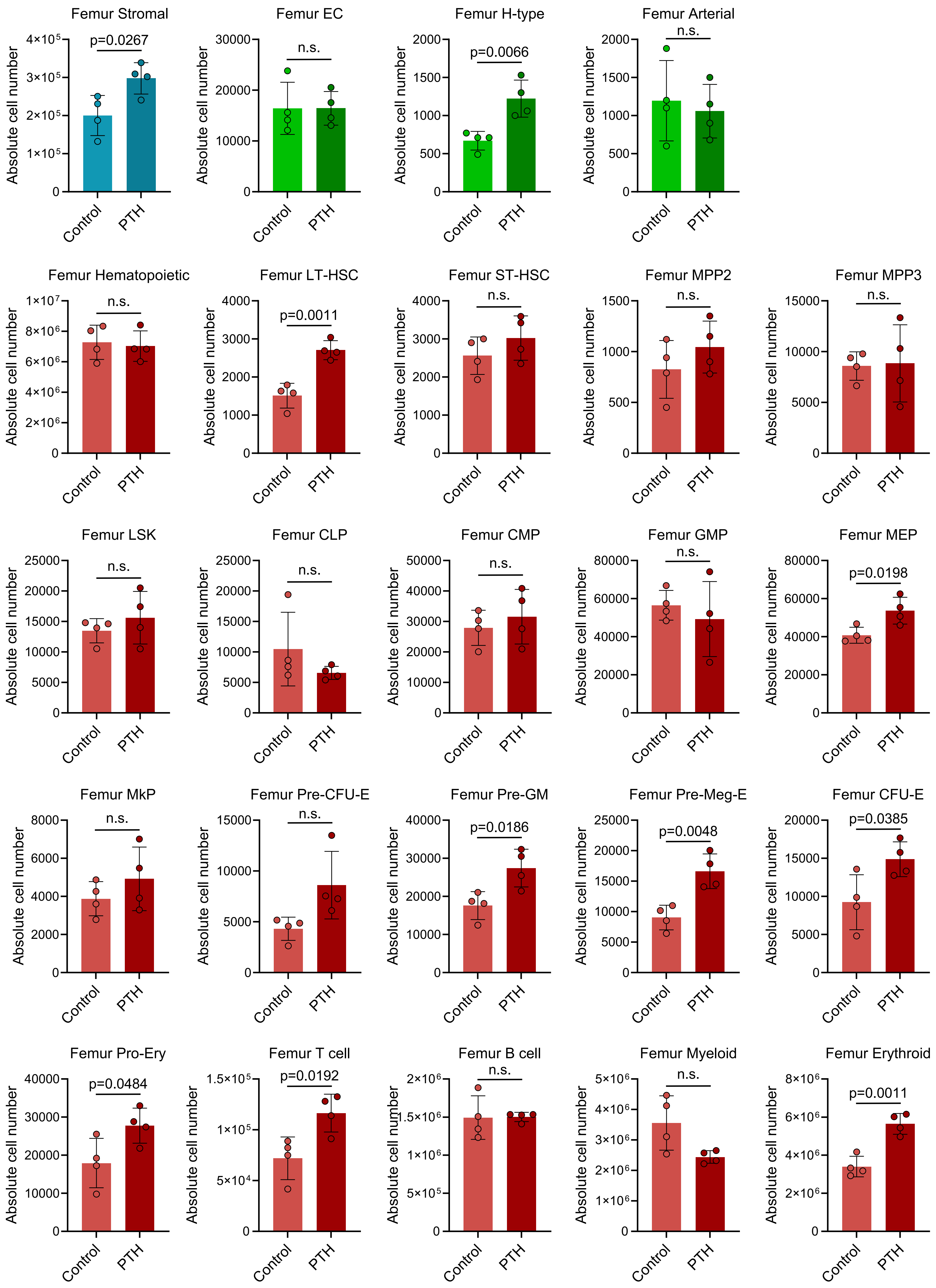

Flow Cytometric Analysis of Skull BM after Young *versus* Old Donor Transplantation

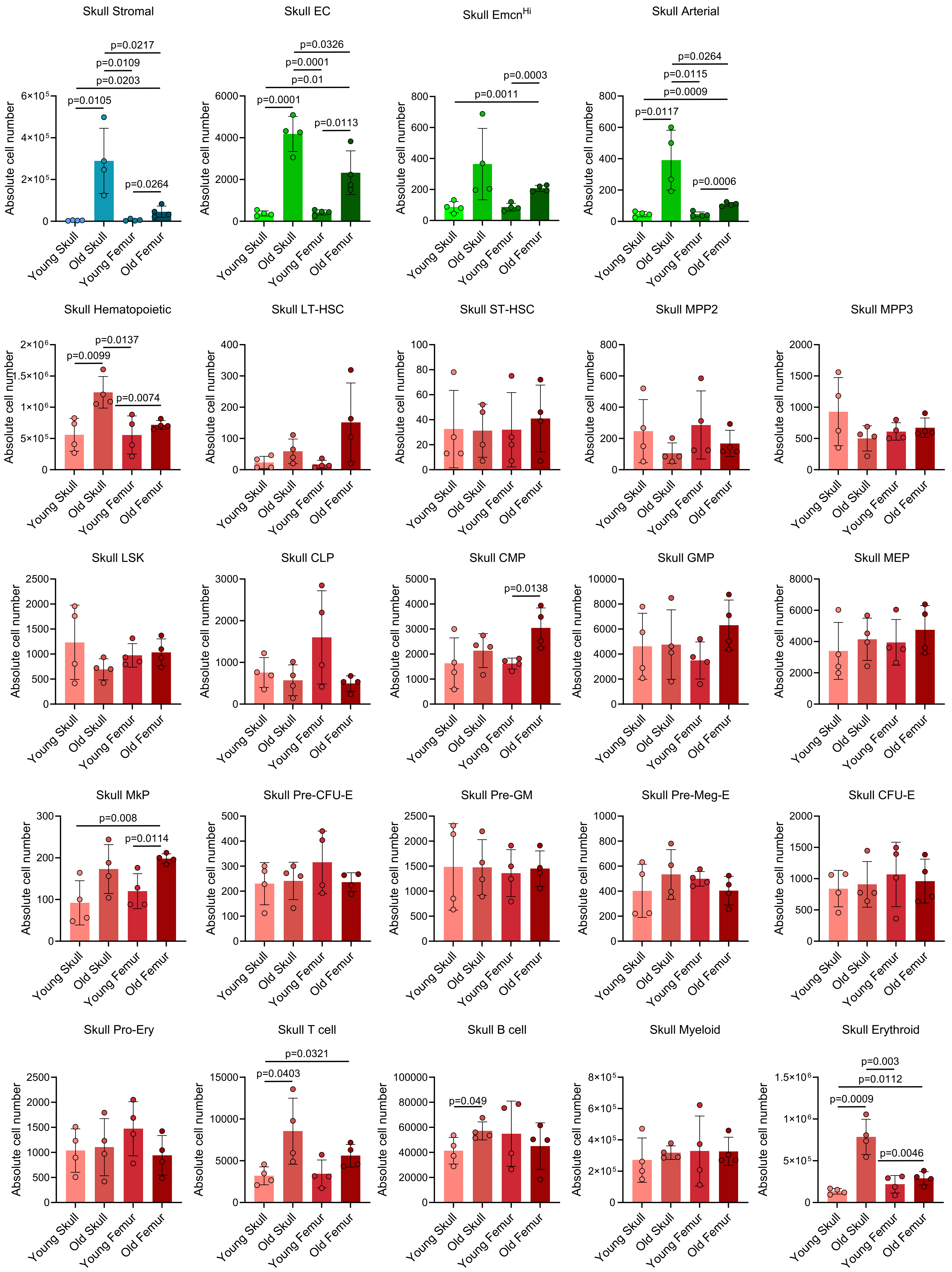

Flow Cytometric Analysis of Femur BM after Young *versus* Old Donor Transplantation

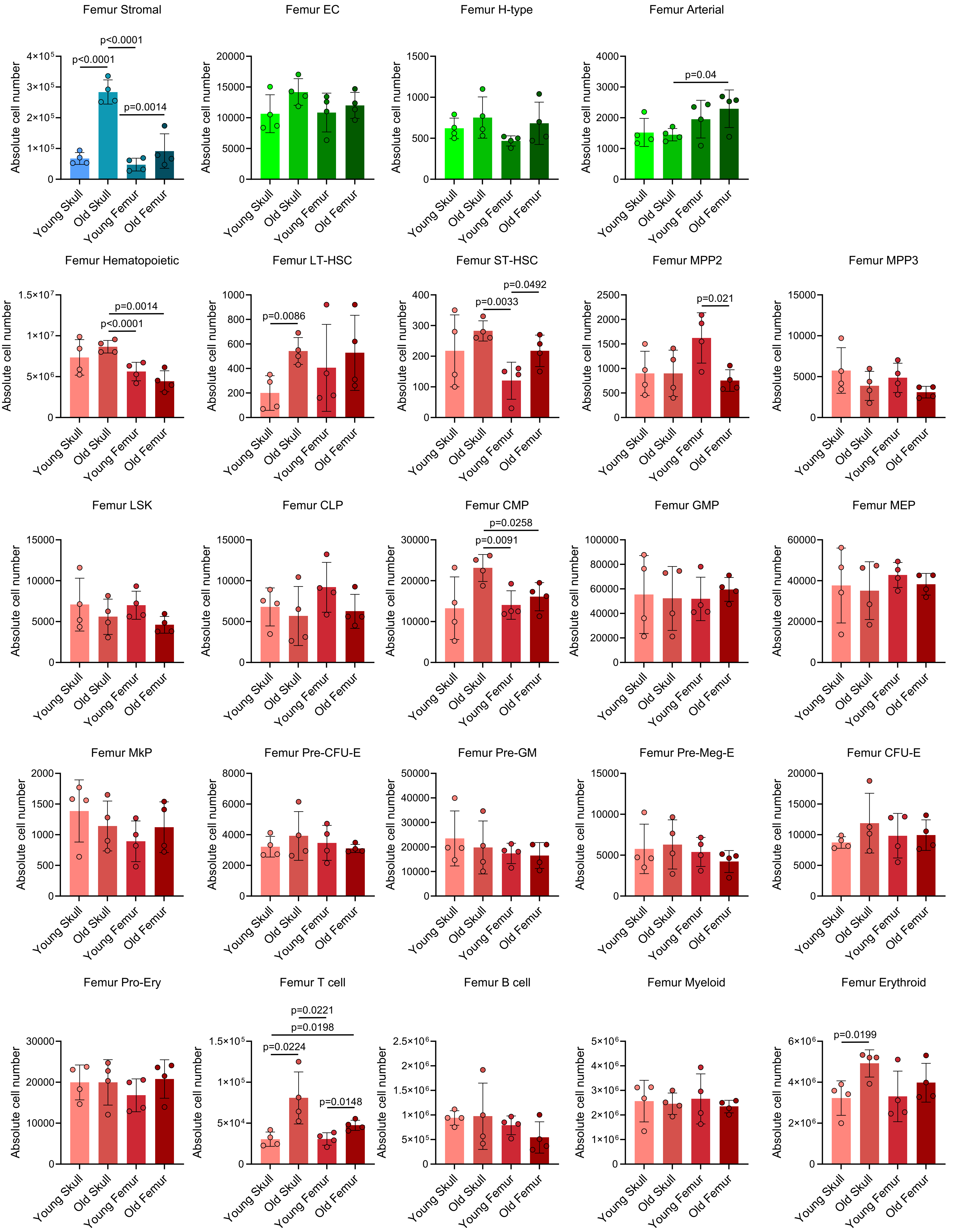

Flow Cytometric Analysis of Skull BM after prostaglandin E2 (PGE2) Treatment

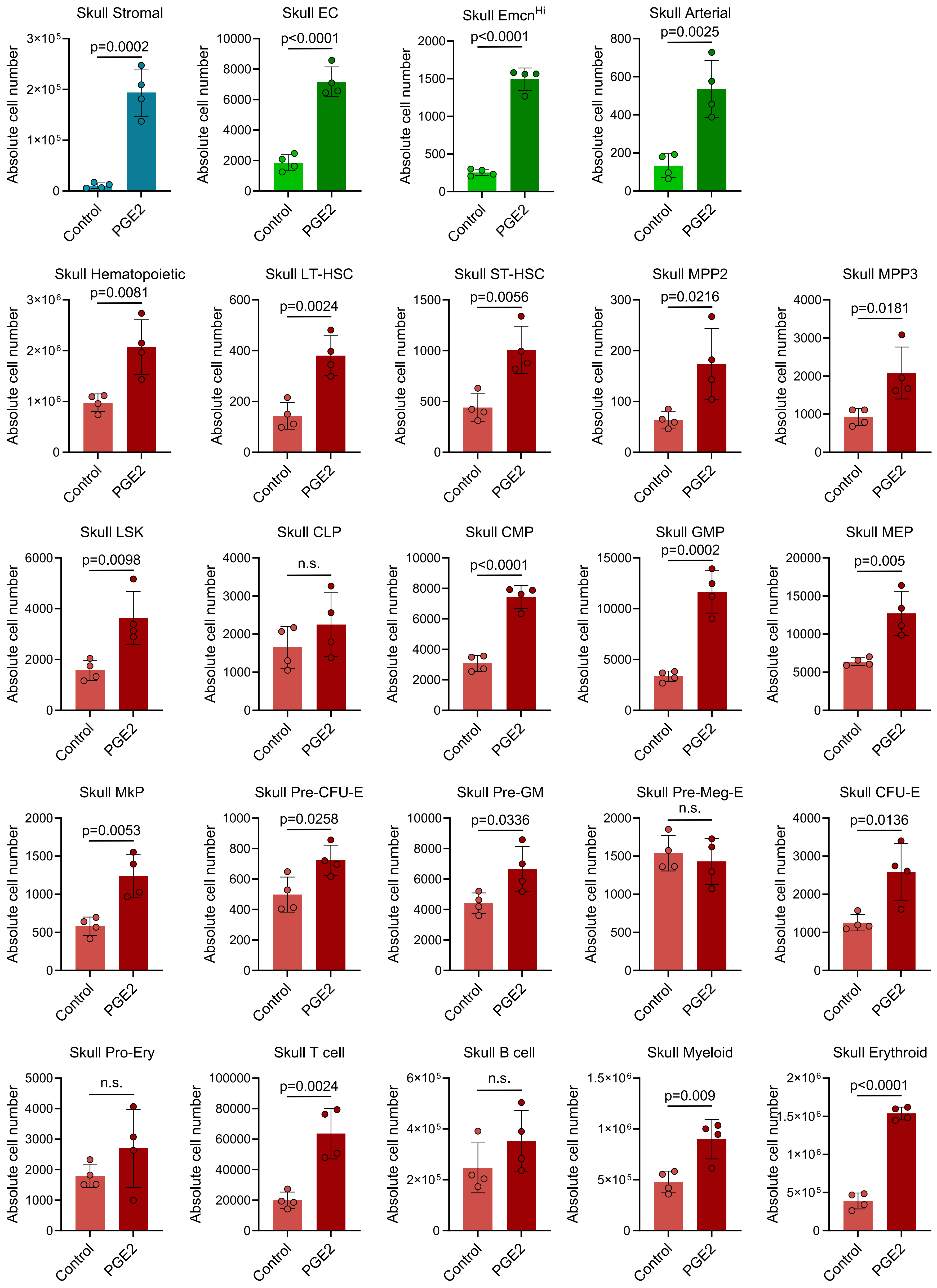

Flow Cytometric Analysis of Femur BM after prostaglandin E2 (PGE2) Treatment

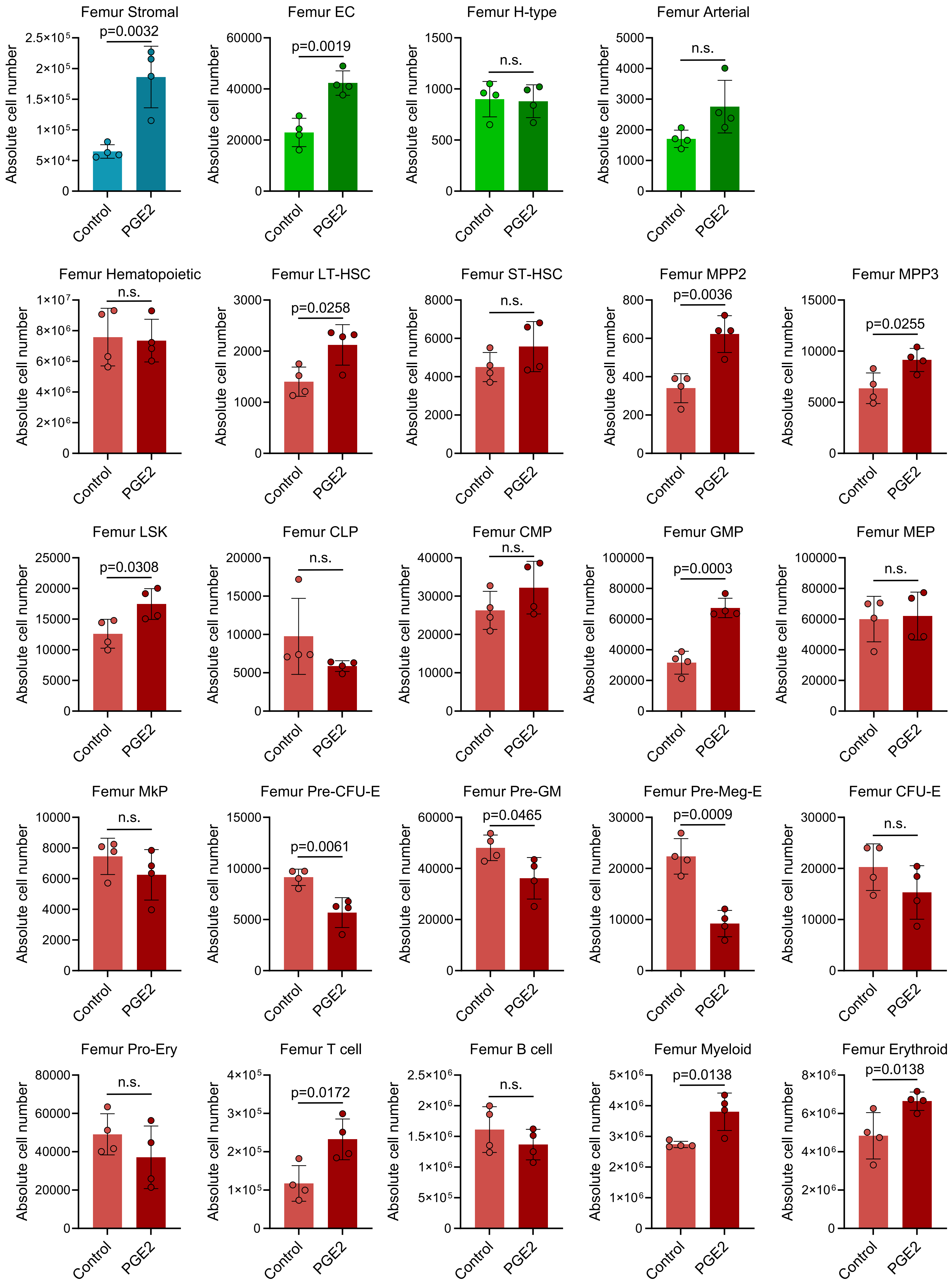

Flow Cytometric Analysis of Skull BM after AMD3100 Treatment

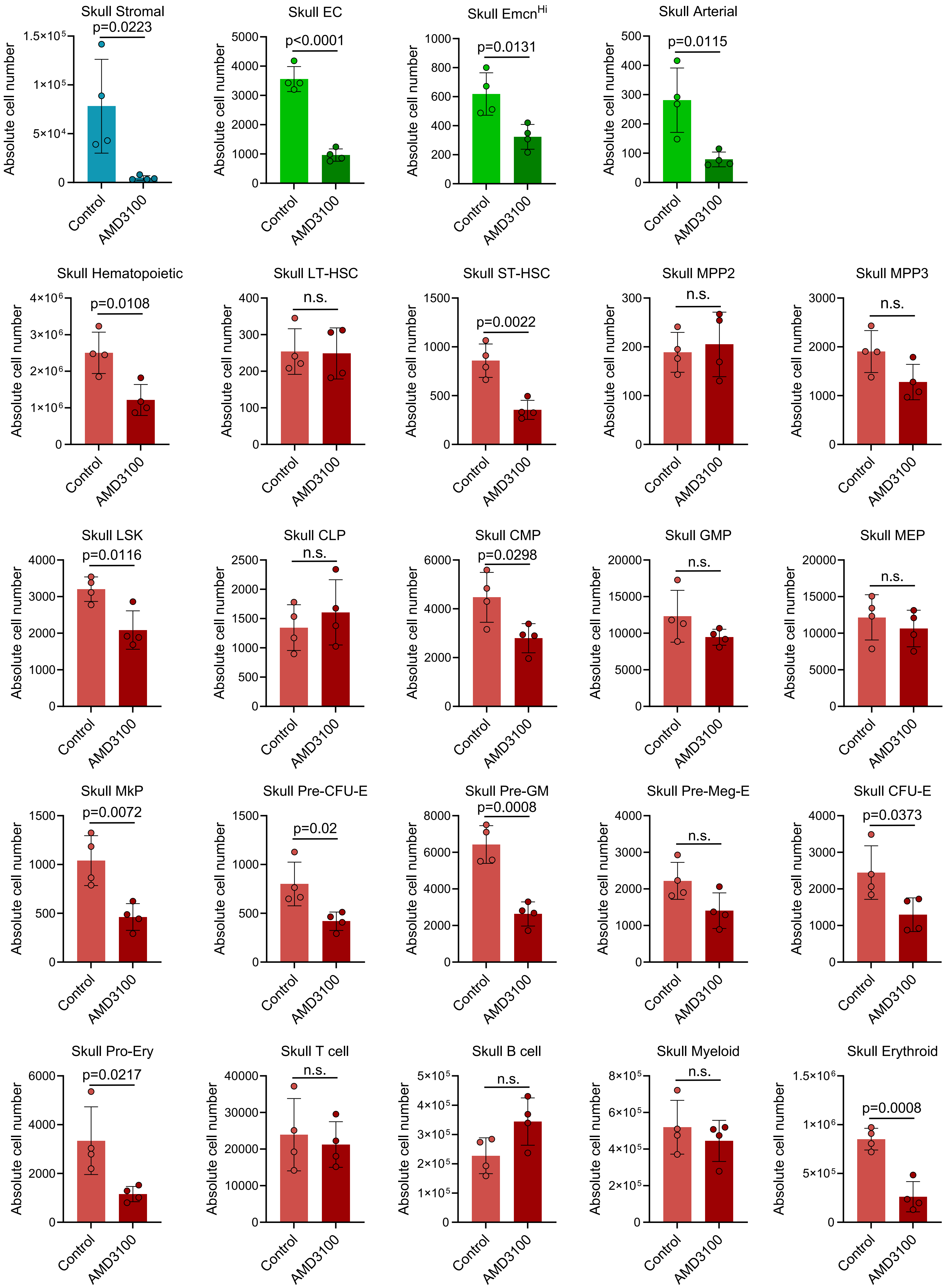

Flow Cytometric Analysis of Femur BM after AMD3100 Treatment

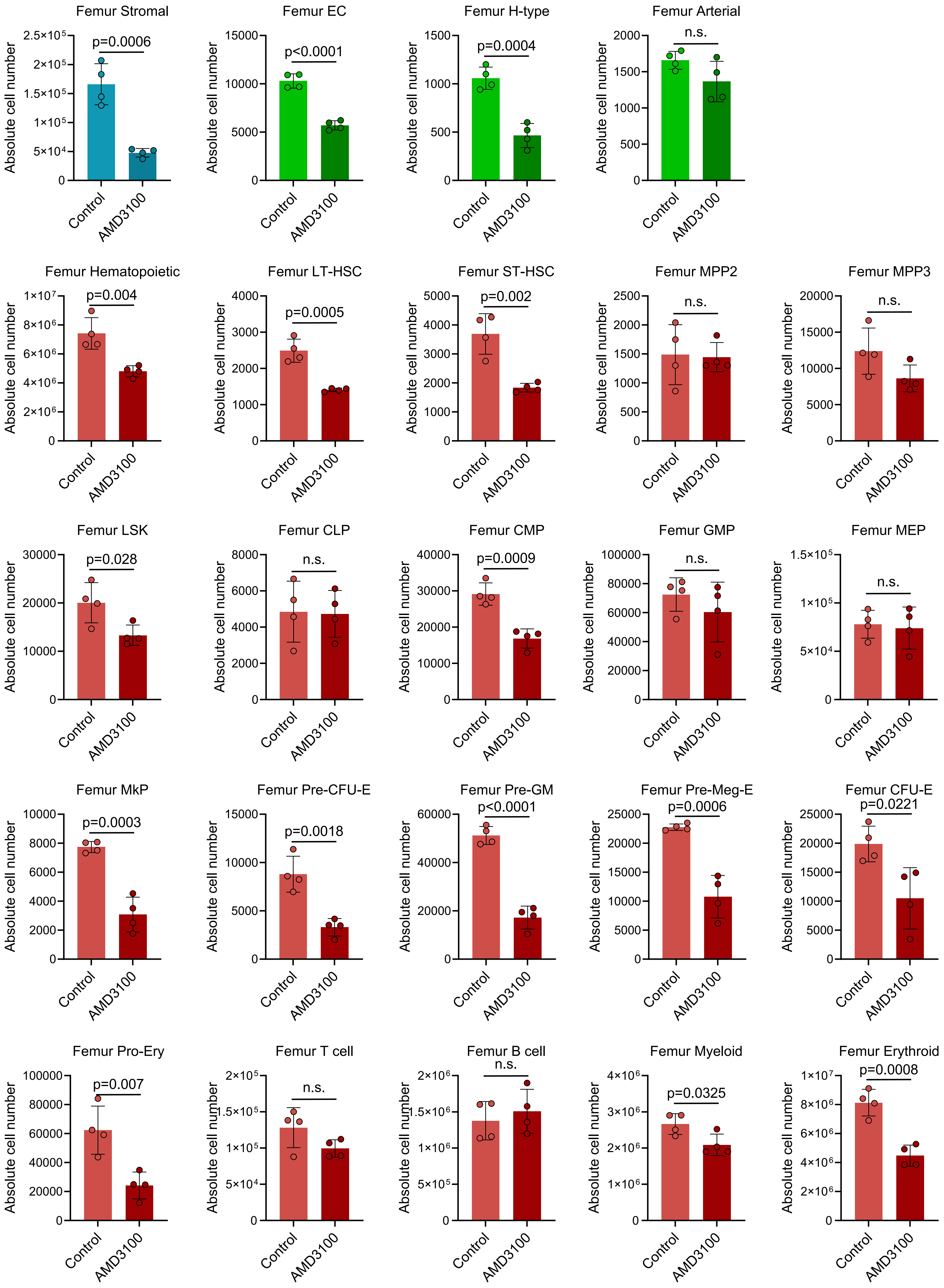

Flow Cytometric Analysis of Skull BM with *pLIVE-Vegfa* Overexpression

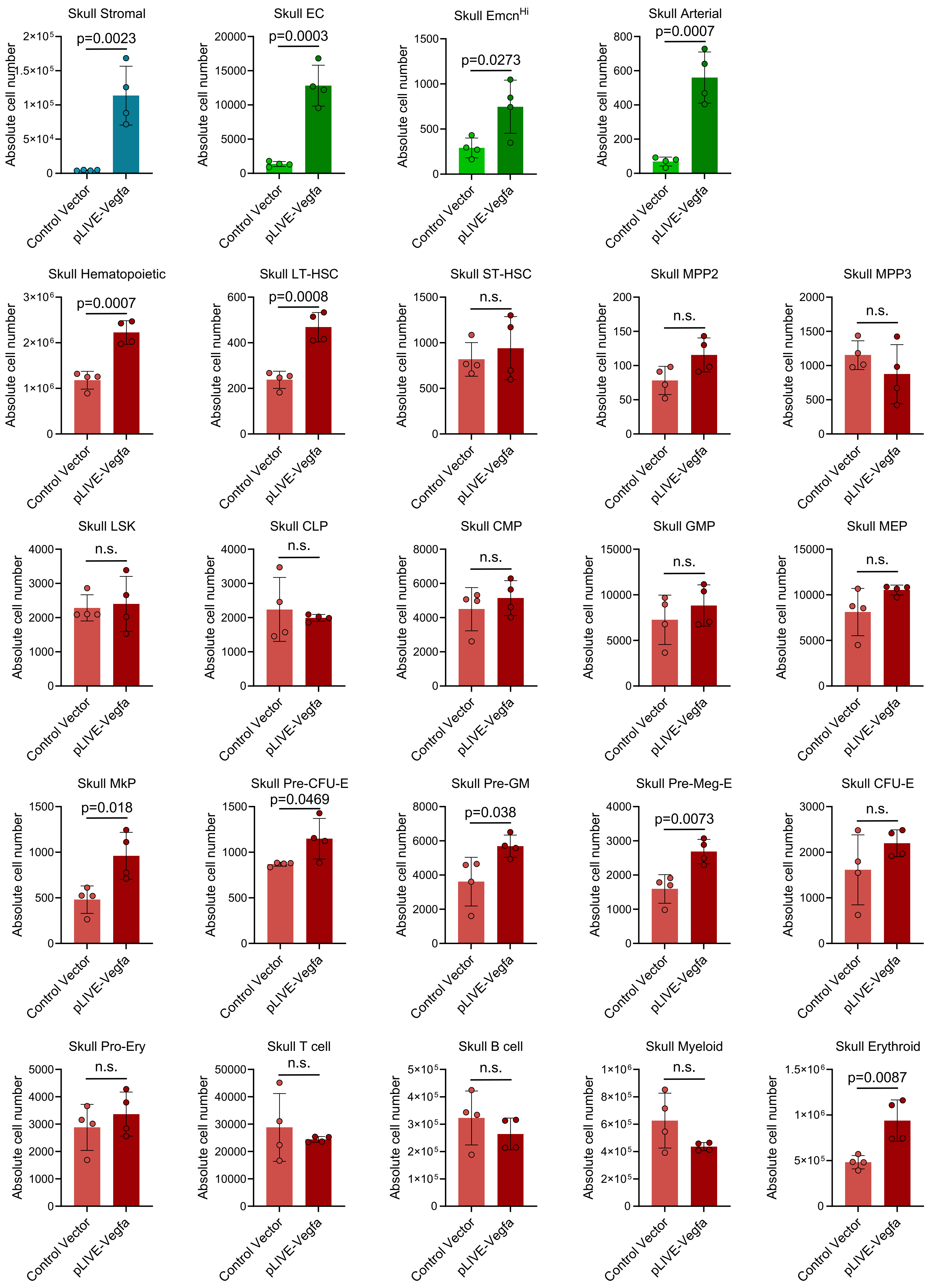

Flow Cytometric Analysis of Femur BM with *pLIVE-Vegfa* Overexpression

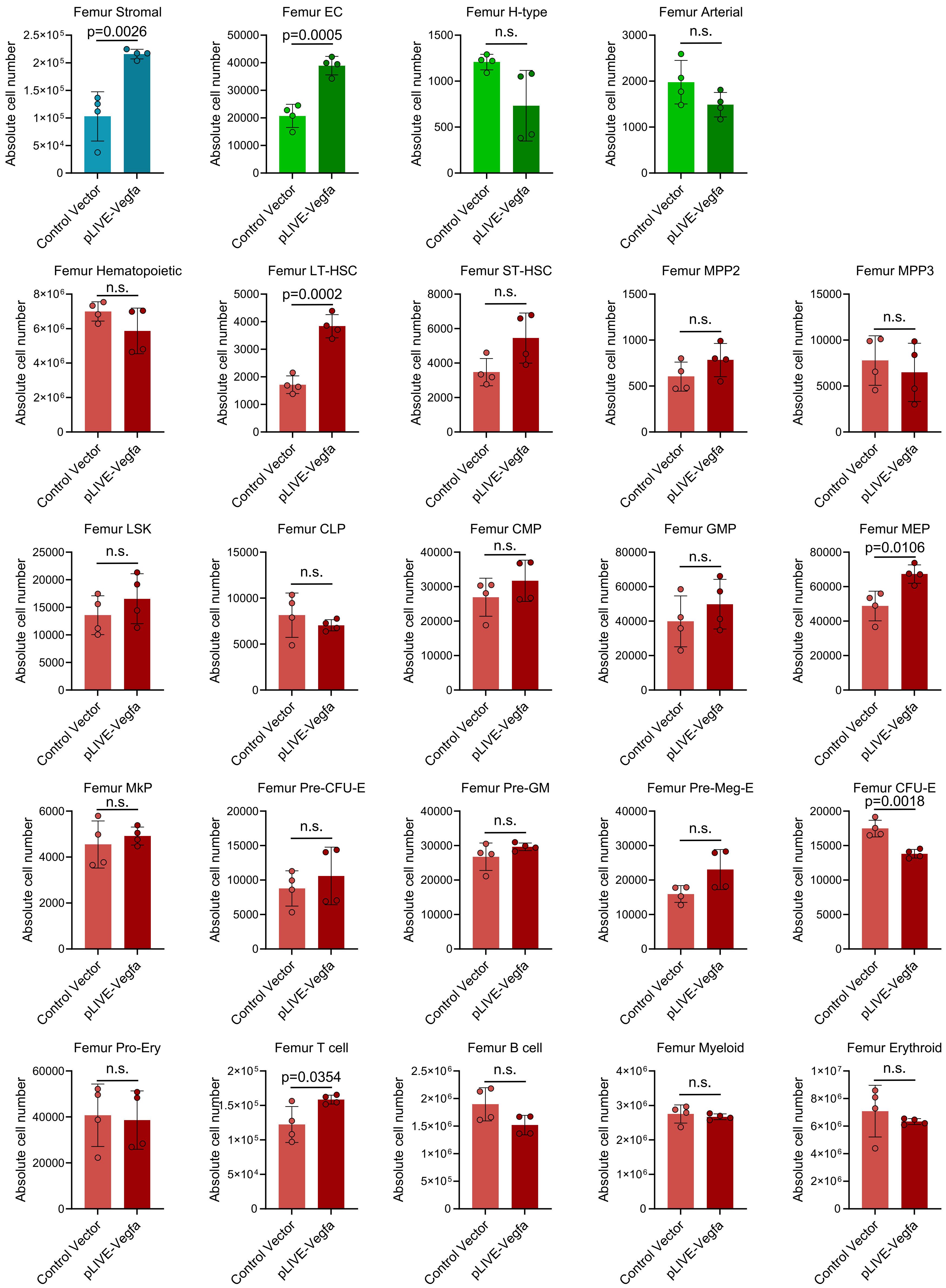

Flow Cytometric Analysis of Skull BM after DC101 Treatment

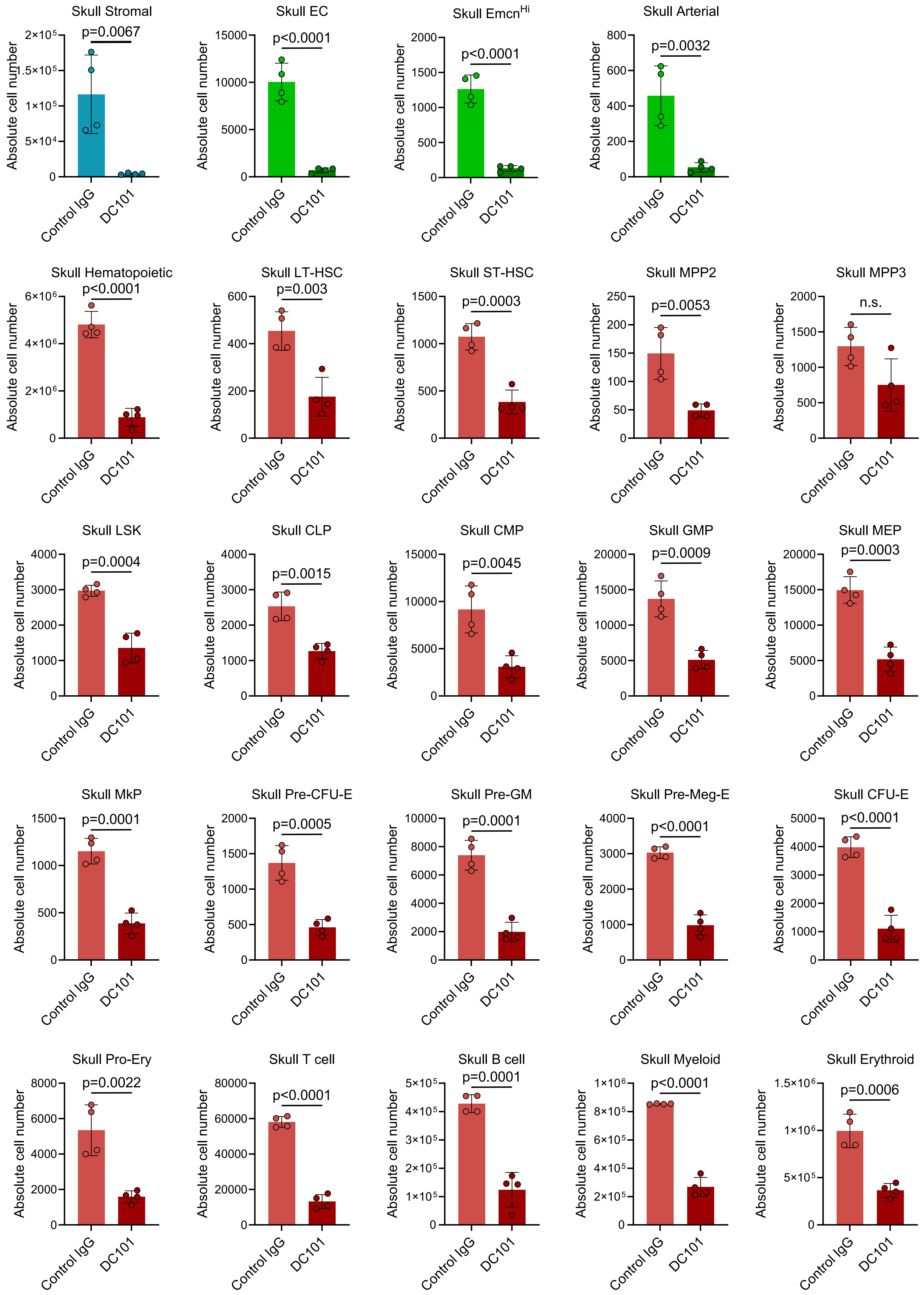

Flow Cytometric Analysis of Femur BM after DC101 Treatment

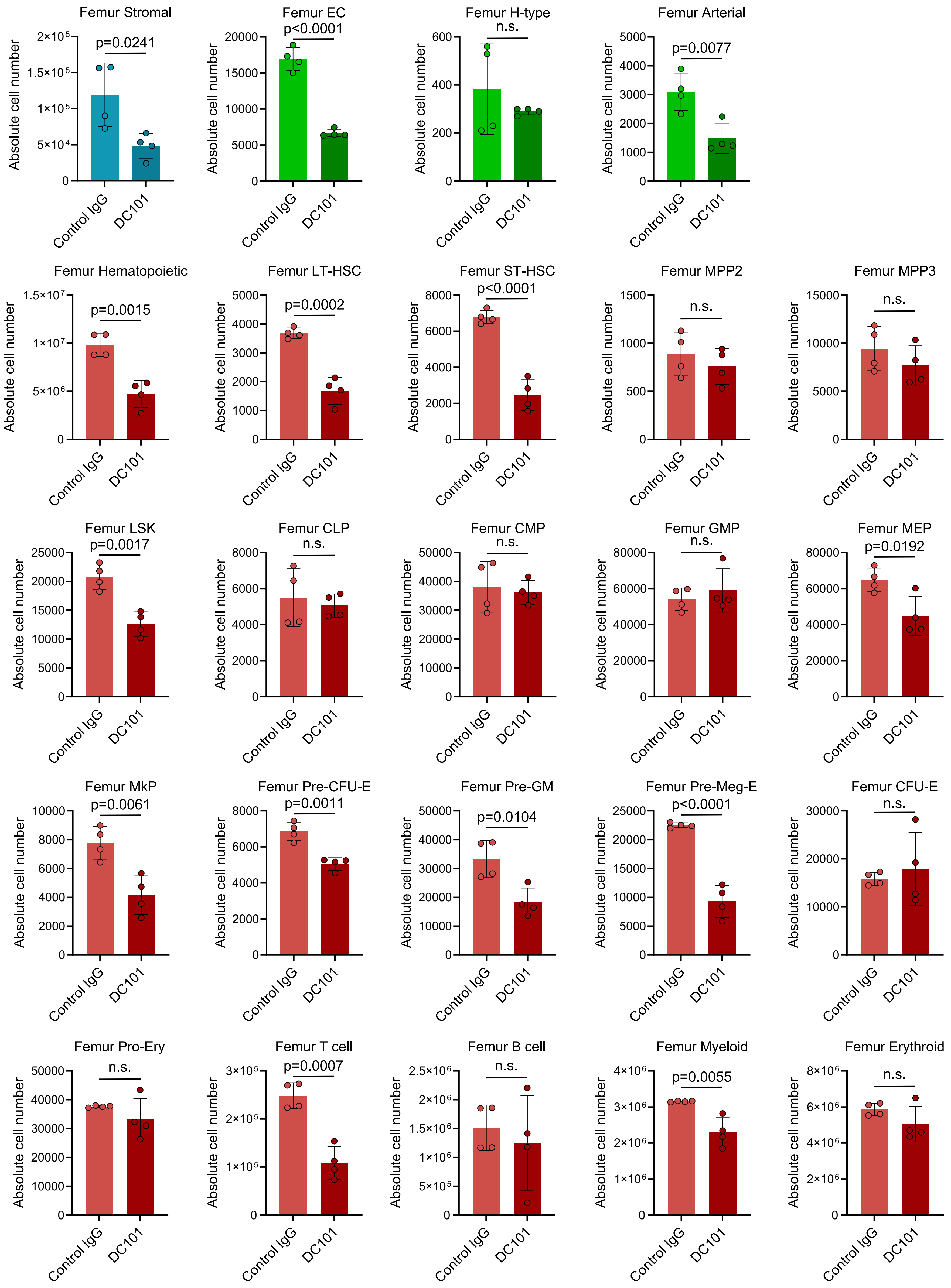

Supplement: Supplementary file 2 — Flow cytometric analyses of skull or femur BM in ageing, pregnancy, stroke, CML, PTH treatment, young versus old transplantation, PGE2 treatment, AMD3100 treatment, pLIVE-Vegfa overexpression and DC101 treatment. [file 41586_2024_8163_MOESM2_ESM.pdf]
