# Supplementary material for: Adult skull bone marrow is an expanding and resilient haematopoietic reservoir
Source: Nature. 2024 Nov 13;636(8041):172–81. doi: 10.1038/s41586-024-08163-9 (PMC11618084; doi:10.1038/s41586-024-08163-9)
Supplement: Supplementary file 3 — Gating strategies for all FACS isolation and flow cytometric analyses performed in the study. [file 41586_2024_8163_MOESM3_ESM.pdf]

Adult skull bone marrow is an expanding and resilient hematopoietic reservoir

Bong Ihn Koh[1,#], Vishal Mohanakrishnan[1], Hyun-Woo Jeong[2], Hongryeol Park[1], Kai Kruse[3], Young Jun Choi[4], Melina Nieminen-Kelhä[5], Rahul Kumar[6], Raquel S. Pereira[7], , Susanne Adams[1], Hyuek Jong Lee[8], M. Gabriele Bixel[1], Peter Vajkoczy[5], Daniela S. Krause[6], Ralf H. Adams[1,#]

Supplementary Data File 2

Gating strategies for all FACS isolation and flow cytometric analyses performed in the study.

Table of contents

a) Representative gating strategy for FACS isolation and flow cytometric analysis of HSPCs

b) Representative gating strategy for flow cytometric analysis of myeloid cells

c) Representative gating strategy for flow cytometric analysis of photo-converted RFP$^+$ cells

d) Representative gating strategy for flow cytometric analysis of hematopoietic progenitors

e) Representative gating strategy for flow cytometric analysis of hematopoietic precursors

f) Representative gating strategy for flow cytometric analysis of lymphocytes and myeloid cells

g) Representative gating strategy for flow cytometric analysis of hematopoietic, stromal and endothelial cells

h) Representative gating strategy for flow cytometric analysis of lymphocytes and myeloid cells in peripheral blood

Adult skull bone marrow is an expanding and resilient hematopoietic reservoir

Bong Ihn Koh[1,#], Vishal Mohanakrishnan[1], Hyun-Woo Jeong[2], Hongryeol Park[1], Kai Kruse[3], Young Jun Choi[4], Melina Nieminen-Kelhä[5], Rahul Kumar[6], Raquel S. Pereira[7], , Susanne Adams[1], Hyuek Jong Lee[8], M. Gabriele Bixel[1], Peter Vajkoczy[5], Daniela S. Krause[6], Ralf H. Adams[1,#]

Supplementary Data File 1

Abbreviations

EC: Endothelial Cell

$Emcn^{Hi}$: Endomucin-High

LT-HSC: Long-Term Hematopoietic Stem Cell

ST-HSC: Short-Term Hematopoietic Stem Cell

MPP2: Multi-Potent Progenitor 2

MPP3: Multi-Potent Progenitor 3

LSK: $Lin^{-} Sca1^{+} cKit^{+}$

CLP: Common Lymphoid Progenitor

CMP: Common Myeloid Progenitor

GMP: Granulocyte-Monocyte Progenitor

MEP: Megakaryocyte-Erythrocyte Progenitor

MkP: Megakaryocyte Progenitor

Pre-GM: Pre-Granulocyte-Monocyte

Pre-CFU-E: Pre-Colony-Forming Unit-Erythroid

Pre-Meg-E: Pre-Megakaryocyte-Erythroid

CFU-E: Colony-Forming Unit-Erythroid

Pro-Ery: Pro-Erythroid

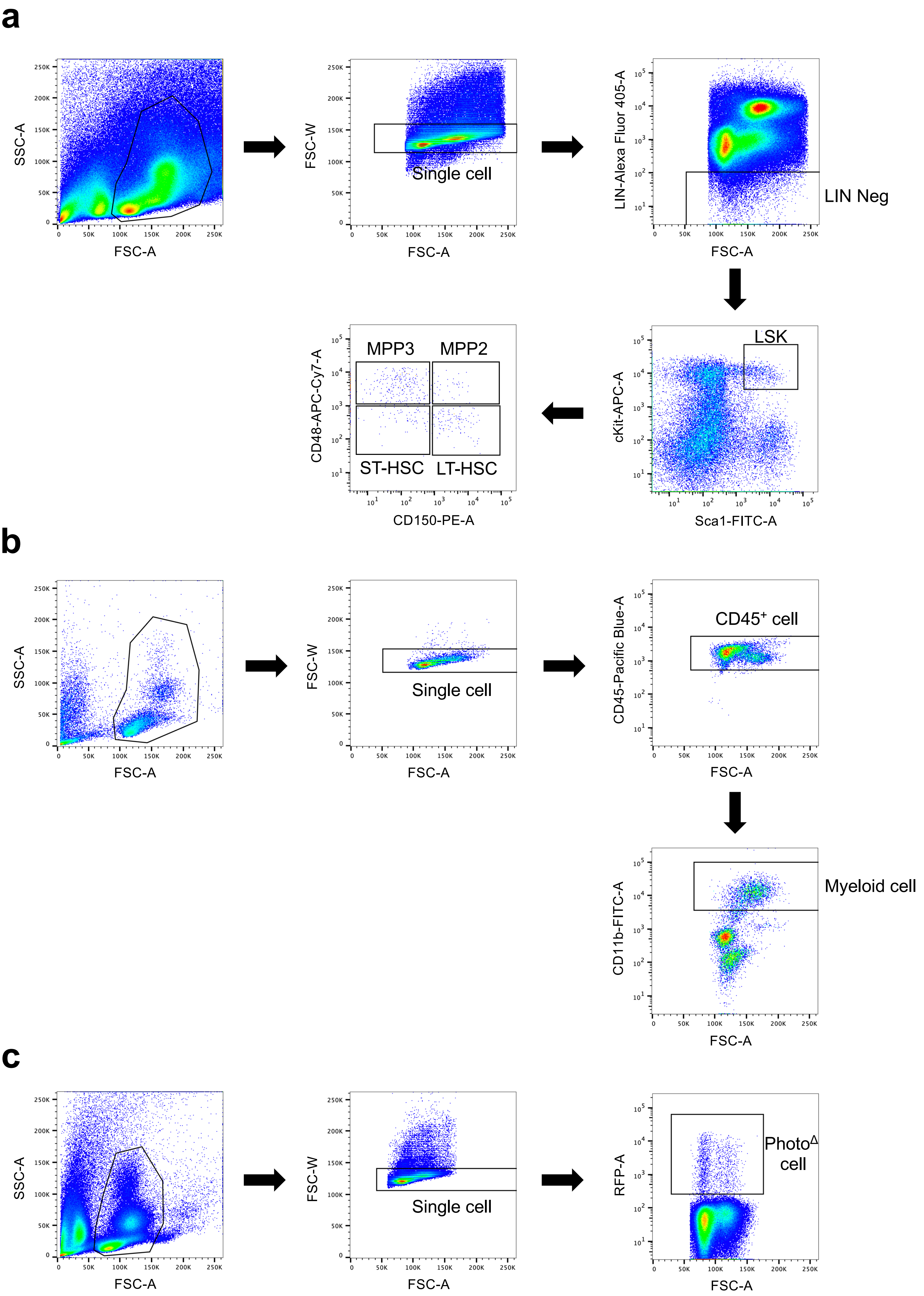

a
SSC-A
FSC-A
FSC-W
Single cell
LIN-Alexa Fluor 405-A
LIN Neg
cKit-APC-A
Sca1-FITC-A
LSK
CD48-APC-Cy7-A
CD150-PE-A
MPP3
MPP2
ST-HSC
LT-HSC
b
SSC-A
FSC-A
FSC-W
Single cell
CD45-Pacific Blue-A
CD45⁺ cell
CD11b-FITC-A
Myeloid cell
c
SSC-A
FSC-A
FSC-W
Single cell
RFP-A
Photo$^{\Delta}$ cell

d

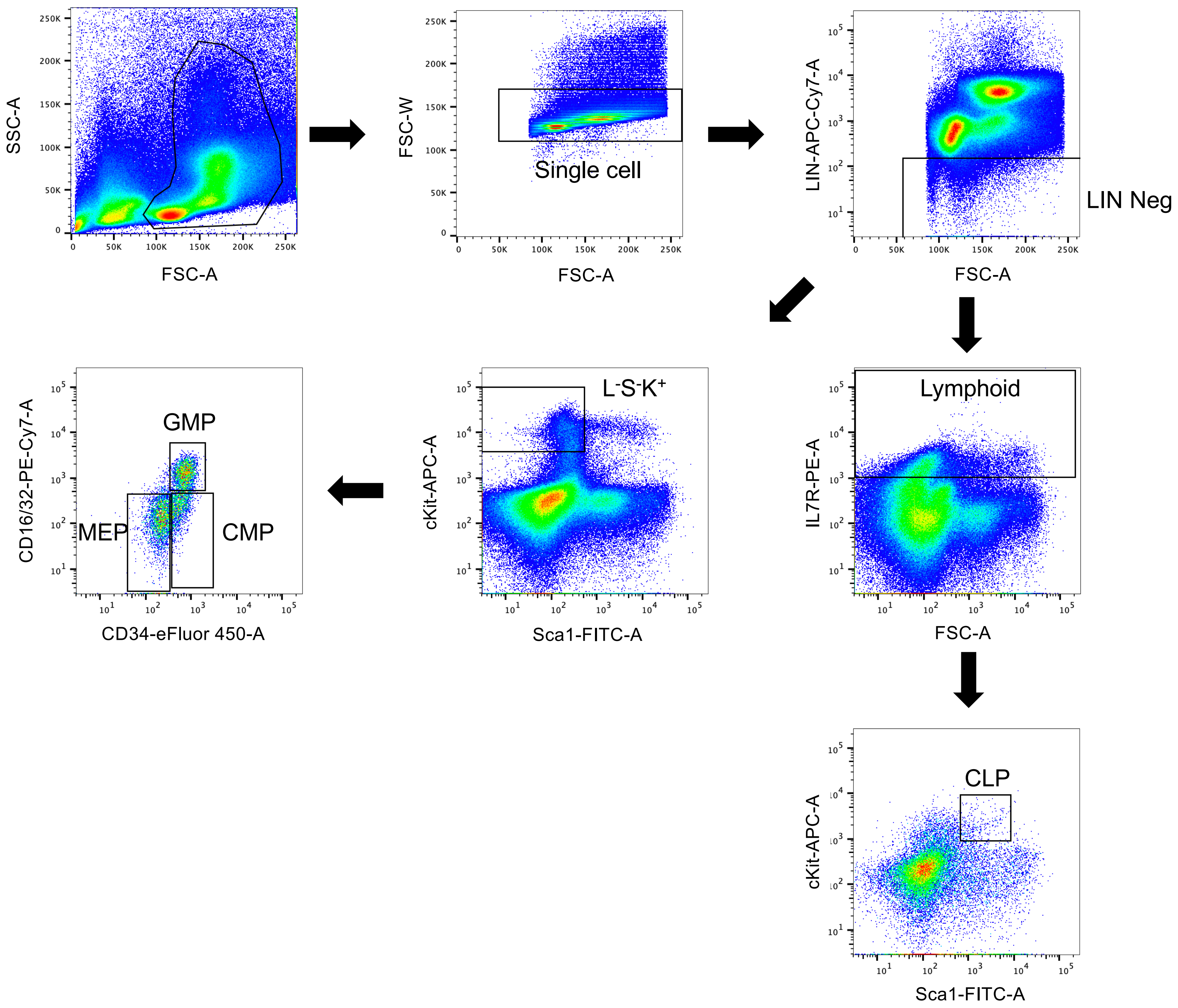

SSC-A
FSC-A
FSC-W
Single cell
LIN-APC-Cy7-A
LIN Neg
CD16/32-PE-Cy7-A
GMP
MEP
CMP
CD34-eFluor 450-A
cKit-APC-A
L-S-K+
Sca1-FITC-A
IL7R-PE-A
Lymphoid
CLP

e

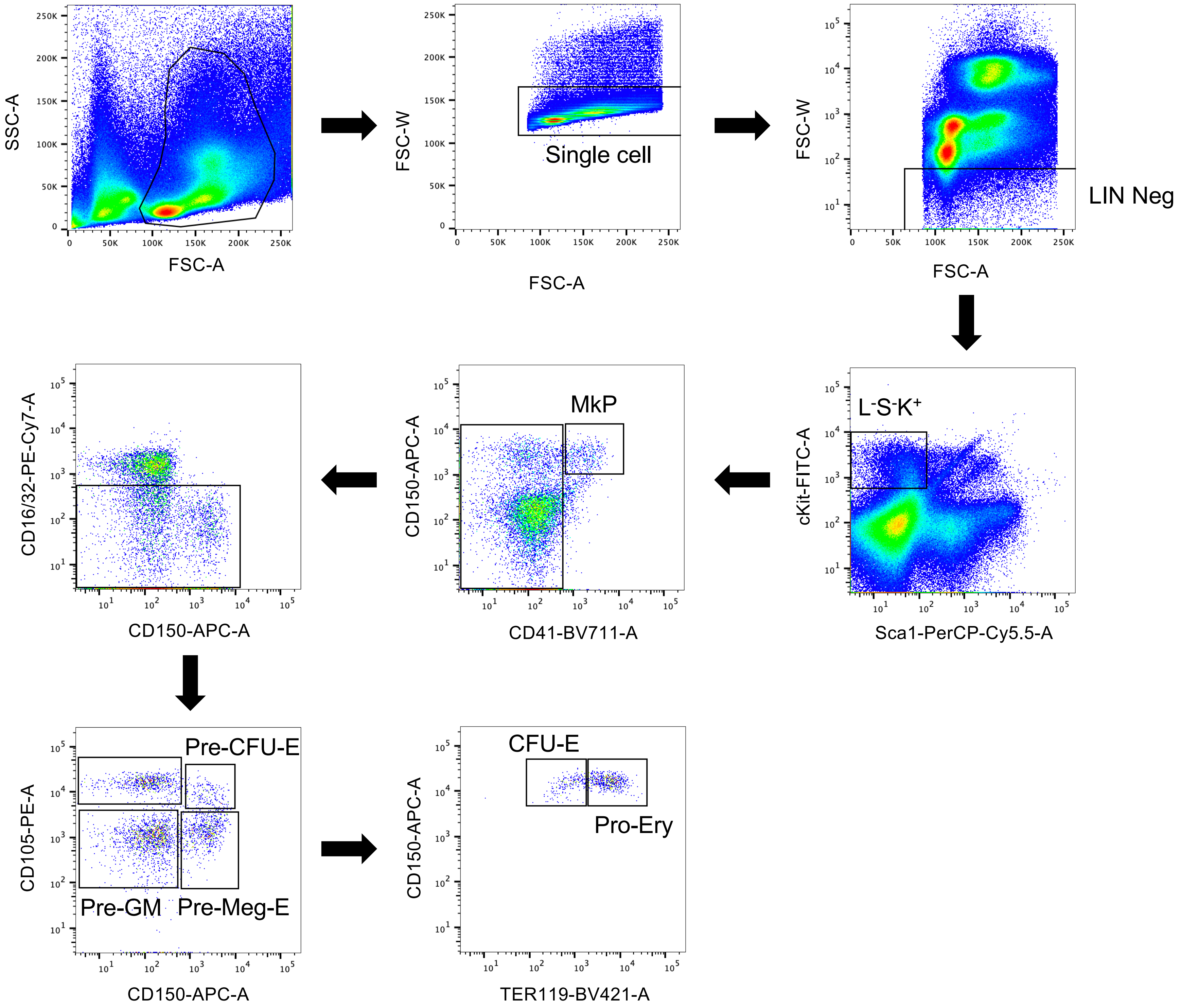
SSC-A
FSC-A
FSC-W
FSC-A
Single cell
FSC-W
FSC-A
LIN Neg
cKit-FITC-A
Sca1-PerCP-Cy5.5-A
L⁻S⁻K⁺
CD150-APC-A
CD41-BV711-A
MkP
CD16/32-PE-Cy7-A
CD150-APC-A
CD105-PE-A
CD150-APC-A
Pre-CFU-E
Pre-GM
Pre-Meg-E
CD150-APC-A
TER119-BV421-A
CFU-E
Pro-Ery

f

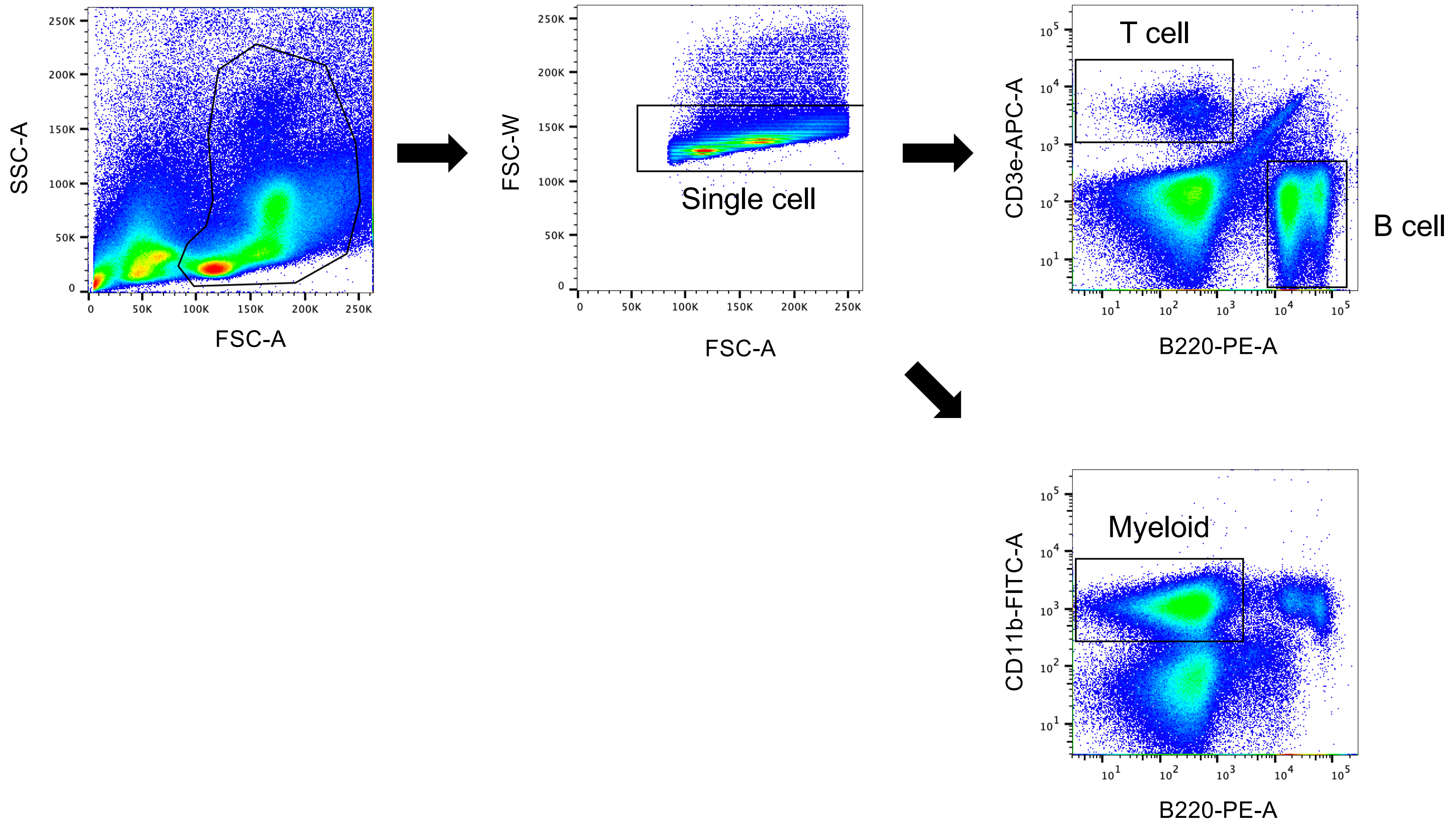
SSC-A
FSC-A
FSC-W
FSC-A
Single cell
CD3e-APC-A
B220-PE-A
T cell
B cell
CD11b-FITC-A
B220-PE-A
Myeloid

g

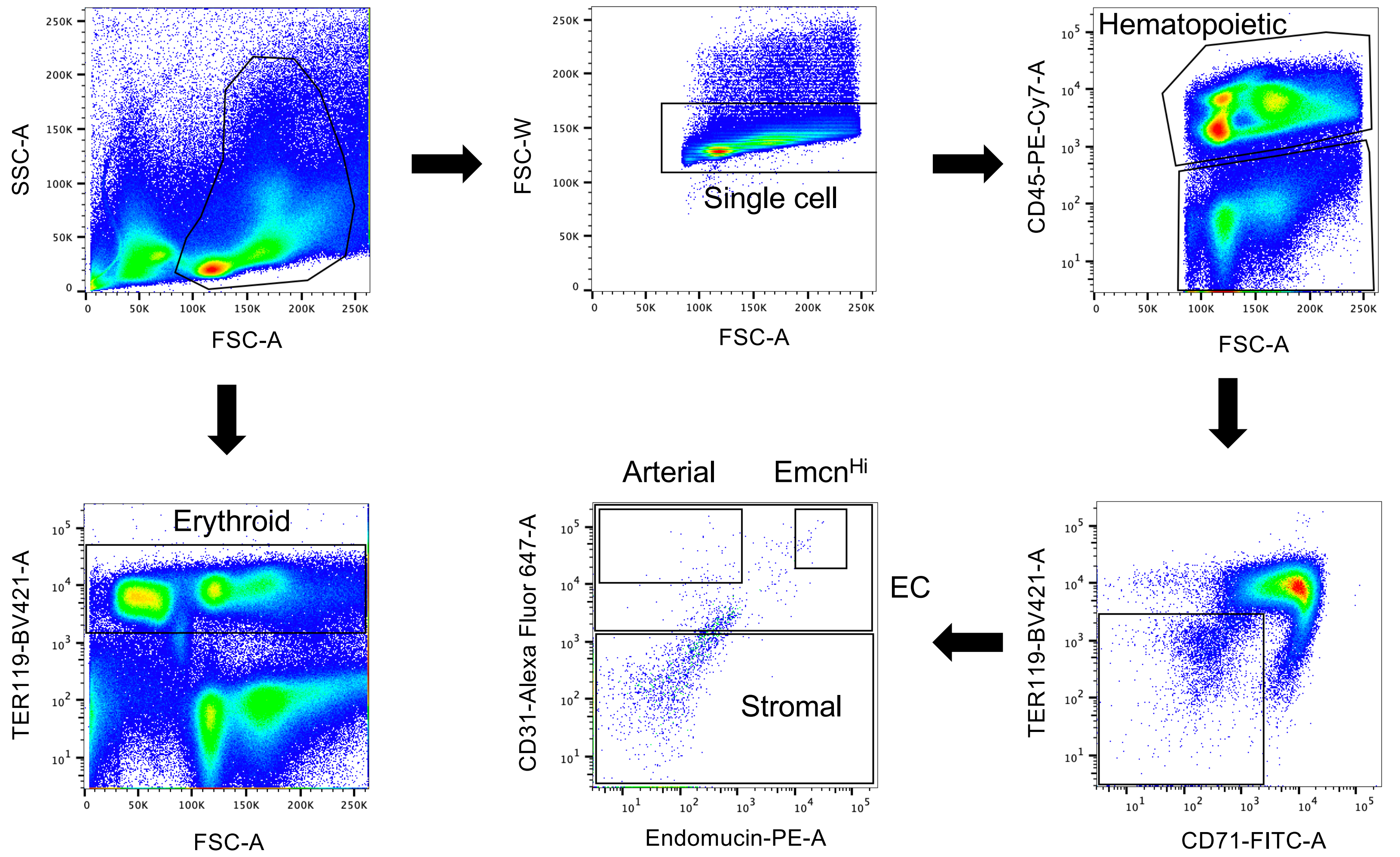
SSC-A
FSC-A
FSC-W
Single cell
CD45-PE-Cy7-A
Hematopoietic
TER119-BV421-A
Erythroid
Arterial
Emcn^Hi
CD31-Alexa Fluor 647-A
EC
Stromal
Endomucin-PE-A
CD71-FITC-A

h

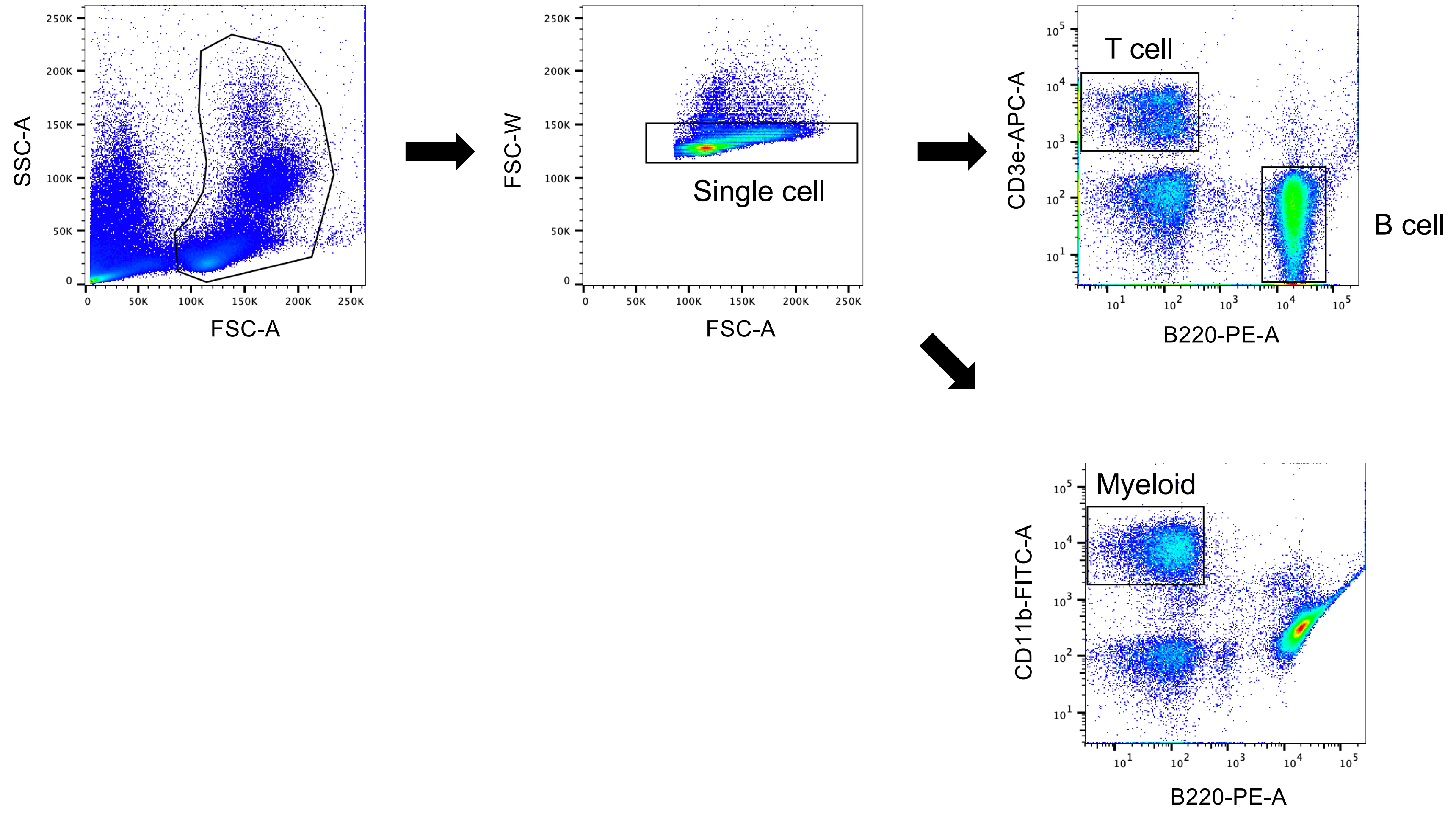
SSC-A
FSC-A
FSC-W
Single cell
CD3e-APC-A
T cell
B cell
B220-PE-A
CD11b-FITC-A
Myeloid
